# Adolescents’ Motivational Profiles in Mathematics and Science: Associations With Achievement Striving, Career Aspirations and Psychological Wellbeing

**DOI:** 10.3389/fpsyg.2019.00990

**Published:** 2019-06-27

**Authors:** Helen M. G. Watt, Micaela Bucich, Liam Dacosta

**Affiliations:** School of Education and Social Work, The University of Sydney, Sydney, NSW, Australia

**Keywords:** expectancy-value, profiles, mathematics, science, motivations, costs, aspirations, psychological wellbeing

## Abstract

Pertinent to concern in Australia and elsewhere regarding shortages in STEM fields, motivational expectancies and values predict STEM study and career aspirations. Less is known about how “cost” values may deter, and how expectancies/values and costs combine for different profiles of learners to predict achievement aspirations and psychological wellbeing outcomes. These were the aims of the present study using established measures of perceived talent, intrinsic and utility values, and a new multidimensional “costs” measure as the platform to explore a typology of mathematics/science learners. Grade 10 Australian adolescents (*N* = 1,172; 702 girls) from 9 metropolitan Sydney/Melbourne schools completed surveys early 2012/2013. Latent profile analyses educed profiles within each of mathematics and science: “Positively engaged” scored high on positive motivations, low on costs; “Struggling ambitious” were high for both positive motivations and costs; “Disengaged” exhibited generally low scores on positive motivations but high costs. MANOVAs examined mathematics/science profile differences on clustering variables, experienced learning environments, achievement background and striving, career aspirations and psychological wellbeing. Positively engaged/Struggling ambitious were distinguished by high costs perceived by Struggling ambitious, associated with debilitated psychological wellbeing, but not eroding achievement striving. A greater proportion of boys was in this risk type. Disengaged students reported lowest STEM-related career aspirations, aimed marks and history of results; in mathematics, a greater proportion of girls was in this risk type. Profiles could be conceptualized along dimensions of achievement striving and psychological wellbeing. Similar profiles for mathematics and science, and coherent patterns of antecedents and outcomes, suggest several theoretical and educational implications.

## Introduction

There is concern, in Australia and elsewhere, regarding shortages in “STEM” fields (science, technology, engineering and mathematics). STEM skills contributed 65% toward Australia’s economic growth between 1964 and 2005 and are required by three-quarters of the fastest growing occupations (Office of the Chief Scientist [OCS], 2014a). Global shortages of STEM capable workforces are predicted to worsen and impact economic and social development (Organisation for Economic Co-operation and Development [OECD], 2006; Office of the Chief Scientist [OCS], 2014b). Over the past two decades, STEM participation has declined in many western nations, including Australia, where there has been a concerning trend away from high school advanced mathematics, physics and chemistry (Federation of Australian Scientific and Technological Societies [FASTS], 2002; Ainley et al., 2008; Dobson, 2012). Women are less likely to choose STEM careers, and more likely to leave once entered (NSCRC, 2013; Cheryan et al., 2017). Adolescence is the crucial time when most students make choices whether to concentrate on STEM in the future (Maltese and Tai, 2011), when course selections can foreclose future educational and career pathways (Watt, 2006). In fact, much of the disparity in both gender and ethnic representation in STEM fields at university could be accounted for by differences in secondary school course-taking (Riegle-Crumb and King, 2010). Although convenient to refer collectively to “STEM”, this can mask different patterns between, as well as within those fields (e.g., physics, chemistry, and biology within science). Our study examines each of mathematics and science as core curricula studied until the end of grade 10 in Australia, after which students choose their courses for upper secondary school.

### Theoretical Framework

Eccles et al.’s (1983) expectancy-value theory (EVT; Eccles, 2005) offers a comprehensive framework to explain achievement-related choices. Initially developed to explain gender differences in high school mathematics enrolments, EVT has become a foremost motivation framework to understand how youths’ beliefs predict educational choices (Jacobs and Simpkins, 2005). At its core, the model posits that expectancy-related beliefs interact with different kinds of values, to predict achievement behaviours and choices. Expectancies and values are contextualised in a developmental framework drawing on decision, achievement goal, attribution and self-worth theories to provide an integrated framework accounting for origins stemming from childhood. The components of task value include intrinsic value or interest, attainment value (referring to the personal importance of doing well), utility value, and cost. Most studies have focused on the first three positive values, at times combining attainment and utility values into an “importance value”; some researchers have measured additional value factors (e.g., Gaspard et al., 2015 distinguished 11 task values). A wealth of studies has collectively established that expectancy-related beliefs (e.g., perceived competence, perceived talent, self-concept) and intrinsic/utility/importance values predict achievement-related choices, including enrolments and career aspirations (for reviews see Watt, 2010, 2016; Wigfield and Cambria, 2010).

The bulk of literature has been in relation to mathematics because it was identified as a “critical filter” (Sells, 1980) limiting girls’ and women’s access to certain high-status and high-income fields of education and career. Gendered mathematics values and ability beliefs predict advanced participation over and above achievement background, for mathematical enrolments (Updegraff et al., 1996; Simpkins et al., 2012; Watt et al., 2012) and career aspirations (Watt et al., 2012), as well as science enrolment intentions (Ethington, 1991; Atwater et al., 1995), scientific career aspirations (Watt et al., 2017a, b), and pursuit of a science career in adulthood (Farmer et al., 1999). Science expectancies and values (including costs) predicted planned completion of a STEM major among college students (Perez et al., 2014).

Researchers within this framework have recently targeted the negative cost value for empirical attention. From the outset (Eccles et al., 1983; Eccles, 1987), Eccles described cost in terms of all the negative aspects of engaging in a task including emotional states, such as anxiety, and the amount of effort required in order to succeed, which can impact opportunities to pursue other valued activities (for a review see Wigfield and Eccles, 1992). She posited that positive values (such as interest and enjoyment) should interact with perceived costs to impact task choices, in increasingly complex ways as the choices between different activities become more numerous with age. Wigfield and Eccles (1992) emphasised the importance of examining how values *work together* in influencing students’ task choices and highlighted the conflict that may occur when values are not in synchrony. Although studies have not yet investigated these factors altogether, task-specific asynchronous profiles, in which positive values but also costs are high, would seem likely to exert deleterious effects on general psychological wellbeing.

Until recently, there had been little empirical attention to the measurement of costs within EVT. First studies included the single facet of opportunity cost (Conley, 2012; Trautwein et al., 2012), single factors which mixed facets together (Luttrell et al., 2010; Jiang et al., 2018), a combination of single and mixed factors (opportunity cost versus other mixed costs, Gaspard et al., 2017), or were unable to discern theorised multiple dimensions resulting in an omnibus cost factor (Battle and Wigfield, 2003). In each of those studies, cost was factorially distinct from and inversely correlated with positive task values. Cost negatively predicted planned (Battle and Wigfield, 2003) or actual (Luttrell et al., 2010) enrolments; and explained variance additional to self-efficacy and an aggregate task value factor on disorganisation, procrastination and avoidance intentions, even when controlling for pre-test construct scores (Jiang et al., 2018).

Battle and Wigfield’s (2003) study was conducted among female college students regarding intentions to attend graduate school, rather than among school students in mathematics as were the other studies. Building on their groundwork, Perez et al. (2014) adapted items designed to tap each of effort cost, psychological cost, and opportunity cost among college students in STEM, and were able to confirm their theorised structure. Each cost factor positively predicted intentions to quit a STEM major, most strongly for effort cost. It is this scale that forms the basis for the multidimensional cost measure developed for the present study, adapted from the college context to suit adolescents. A similar goal was expressed by Flake et al. (2015), despite their studies being conducted among college students, who proposed an additional fourth “outside effort” cost (example item: “I have so many other responsibilities that I am unable to put in the effort that is necessary for this class”). However, their factors showed extreme correlations: from 0.83 between outside effort and emotional costs, up to 0.95 between effort and opportunity costs (that they termed “loss of valued alternatives”), with a median correlation of 0.87. Costs inversely correlated with general expectations for success, an omnibus positive value factor, items tapping long-term interest, a single “overall motivation” item, and a final course grade in the college-level calculus course. A measure sensitive to the nuances of different costs is required to allow empirical examination of the tenets of expectancy-value theory with regard to how all values, including costs, work together and with expectancies, to influence choices before students already self-select into their chosen fields.

### Motivation Profiles in the Expectancy-Value Framework

Although expectancies and values are posited to interactively predict choices, many studies have adopted main-effects variable-centred models. Little is known about how expectancies and positive/negative values combine for different profiles of learners, with implications for achievement striving, career aspirations or psychological wellbeing. Latent profile analyses allow the exploration of types of individuals having distinct motivation profiles, to link them with potential antecedents and outcomes. Such person-centred analyses focus on the individual as the unit of analysis, consistent with modern developmental theory (Magnussen, 2000). This approach was adopted in the present study based on the premise that different configurations of expectancies and values (including costs) should impact students’ achievement striving, career aspirations, and potentially psychological wellbeing outcomes.

A small but growing literature has begun to adopt a typological approach informed by EVT constructs. These have either been concentrated in STEM domains (Conley, 2012; Lazarides et al., 2016a, 2018, 2019; Andersen and Chen, 2016; Chittum and Jones, 2017; Linnenbrink-Garcia et al., 2018; Perez et al., 2019), assessed at a domain-general level (Roeser et al., 1999; Tuominen-Soini et al., 2011, 2012), or across multiple domains (Viljaranta et al., 2009; Chow and Salmela-Aro, 2011; Chow et al., 2012; Lazarides et al., 2016b; Guo et al., 2018). A number of studies exist which compare motivations across domains using aggregate task values (combining component positive values into a single score) but exclude expectancy or cost measures (Viljaranta et al., 2009; Chow and Salmela-Aro, 2011; Chow et al., 2012; Guo et al., 2018); while others include aggregate values plus self-concept (Lazarides et al., 2016b). Emergent profiles from this body of work characterise relative valuing across subjects (e.g., mathematics and physical science versus English; Chow et al., 2012). While illuminating in their own right, our aim is to identify groups of students *within* each domain of mathematics and science, including the breadth of expectancy and values constructs. We review studies in this section which focused on STEM-specific motivational profiles informed by the EVT framework.

There has been a line of research focused on EVT motivational profiles in mathematics (Lazarides et al., 2016a, 2018, 2019), although not all of them have included multiple value factors and none included costs. Lazarides et al. (2018) found two profiles among Finnish first- and second-graders who showed consistently low or high levels of interest value, self-concept and performance, as well as a group with medium interest despite low self-concept and performance. A fourth group was characterised by low interest, yet medium levels of self-concept and performance. These profiles supported the authors’ expectations that there would be mixed as well as consistent configurations of value and expectancy, although only intrinsic value was included. Lazarides et al. (2016a, b) identified four profiles among German adolescents based on a wider range of values (intrinsic, utility, and attainment) and self-concept: three showed consistent configurations (low, moderate, and high), and a fourth mixed group (containing more girls) showed moderate to high utility value but lower scores on other dimensions. Most recently, using a large sample (*N* = 6,020) of German 9th–10th graders from upper- and middle-track schools (PISA-I Plus study in 2003–2004; Prenzel et al., 2006). Lazarides et al. (2019) identified similar profiles: low, medium, high, and an infrequent mixed type (4% of sample) characterised this time by high self-concept, low interest and pronounced utility value. Similar profiles were identified for science motivation (Andersen and Chen, 2016; Chittum and Jones, 2017; who added teacher caring as a clustering variable). It seems that utility value can be high, independent of other kinds of internal values. Including negatively valenced constructs (i.e., costs) may yield more nuanced, asynchronous profiles.

Only three studies included cost (Conley, 2012; Bøe and Henriksen, 2013; Perez et al., 2019) among clustering variables in examining students’ motivational profiles. Using selected variables from EVT and achievement goal theory, Conley (2012) identified 7 clusters of 7th-grade United States students who could be grouped at “low”, “average”, and “high” levels of mathematics motivation. Within these broad groups, clusters were further distinguished by perceptions of cost (only “opportunity cost” was measured). In the 3 clusters with “average” motivation, only the cluster with higher perceived cost differed on achievement and affective outcomes, showing lower levels of achievement, more negative and less positive affect in mathematics class. This highlights the value of including even just one type of cost in distinguishing types of students. Although increased cost did not lead to more negative affect in mathematics class when comparing two clusters with similarly high levels of task values, including multiple dimensions of cost may provide a more complete picture and better prediction of outcomes by tapping the potential strains among highly motivated students. Similarly to Conley (2012), Linnenbrink-Garcia et al. (2018) included EVT as well as achievement goal theory constructs. Unlike Conley (2012), task value (interest, attainment, and utility) was aggregated, which meant clusters were distinguished more by their achievement goals than patterns of values, and costs were not included.

In a sample of undergraduate students enrolled in gateway chemistry courses at an elite United States university, Perez et al. (2019) identified three profiles based on combinations of science competence beliefs (expectancies), values (attainment, utility, and interest) and two kinds of costs (opportunity and effort; validating their previously developed scale also in this independent sample; Perez et al., 2014). One profile characterised by lowest competence beliefs and values with highest costs was labelled “Moderate All” due to their moderate scores on all variables. Moderate scores on positive motivational variables may be expected for even the least motivated, among students at an elite university who already self-selected into a science major. The other two profiles exhibited higher values and perceived competence, with lower costs. These two profiles differed only in their level of values and effort cost as reflected in their names; “Very High Competence/Values-Low Effort Cost” and “High Competence/Values-Moderate Low Costs.” Interestingly, despite equivalent STEM GPA at the end of their 1st and 4th years, students in the “High Competence/Values-Moderate Low Costs” profile completed fewer STEM courses by the end of both years than those in the “Very High Competence/Values-Low Effort Cost” profile. This highlighted the differentiating role of values, competence beliefs and effort cost (but not opportunity cost) in determining STEM participation among these two more motivated profiles. The “Moderate All” profile fared worst in terms of both STEM GPA and course completion at both timepoints, and, women and underrepresented minorities were more likely to fall into that profile. Perez et al. (2019, p. 19) acknowledged the limitation of only assessing two dimensions of cost and recommended inclusion of psychological cost in future research. We would also expect more variation among students not already self-selected into tertiary STEM studies.

Only one other study explicitly measured cost in terms of the negative aspects related to one educational choice compared with others (Bøe and Henriksen, 2013). While termed “relative cost”, it seemed to tap cost in general, rather than a specific dimension (Perez et al., 2019). Students of physics were asked to retrospectively rate the importance in their choice to study physics, alongside expectancies, interest, attainment and utility values (Bøe and Henriksen, 2013). The three resultant profiles differed on positive motivation variables, but all showed similar low levels of cost, quite possibly because only students who had self-selected into physics (potentially in part due to low perceived cost) were sampled. Our study explored expectancies and values including costs at a timepoint preceding students’ choice of STEM enrolments to overcome this limitation.

Akin to psychological cost, test anxiety was measured along with constructs closely related to EVT such as self-efficacy, competence and task value, among secondary school mathematics and science students in Singapore (Ng et al., 2016). Four clusters were educed: “low” (low on motivational beliefs and anxiety), “high” (high motivational beliefs and anxiety), “good” (high motivational beliefs and low anxiety), and “poor” (lowest motivational beliefs but high anxiety). Only achievement correlates were examined, precluding any directional inferences: low and high groups had moderate academic achievement in those subjects, “good” had the highest, and “poor” had the worst. It is interesting that the asynchronous group (“high”) only had moderate achievement despite high motivation, conceivably related to their high anxiety.

There have been growing efforts to integrate wellbeing and achievement motivation-related constructs altogether when identifying typologies of students. An early study combining motivational and wellbeing variables was conducted by Roeser et al. (1999) who identified profiles of grade 8 students in the United States based on their perceived academic competence and task value (each averaged across mathematics and English to approximate “general” school beliefs; value as an aggregate of component values within EVT) and mental health. Analyses yielded four profiles: “well-adjusted” (positive on all three indicators), “multiple problems” (low on all), “poor motivation” (positive mental health, but low competence beliefs and value), and “poor mental health” (poor mental health, despite high competence beliefs and value). Those authors called for further studies at a domain-specific level, encompassing multiple dimensions of psychological wellbeing.

A recent study also aimed at integrating academic motivation and psychological wellbeing dimensions but did so among 15–16 years old Finnish students (Parhiala et al., 2018), based on four motivation factors (aggregate task value for each of mathematics and literacy, school enjoyment, task-focused behaviour) and four wellbeing factors (school burnout, self-esteem, externalising and internalising behaviours). Five identified profiles included three synchronous (high/average/low) as well as two asynchronous types (low motivations but average wellbeing, average motivation but low wellbeing), highlighting the fact that positive motivations need not accompany psychological wellbeing. Those authors retained domain-specific values (aggregated across interest, importance, and usefulness) for mathematics/literacy, but other motivation factors at the domain-general level (about schoolwork in general, which might not equally apply in all learning domains) and did not tap expectancies or costs. Our review could identify no previous study which taps the breadth of EVT constructs including values, expectancies and different types of cost among youth not yet self-selected into STEM studies.

### Profiles Based in Other Motivational Theories

Four types of students were initially theorised and identified by Covington and Omelich (1991) in their seminal quadripolar model of need achievement, distinguished by their degree of success orientation and failure avoidance along two orthogonal continua. Although aligned with achievement goal rather than expectancy-value theory, their seminal work on motivational types is relevant to those we might expect in our study as the stresses tapped by our measure of psychological cost may function similarly to failure avoidance. Their first two groups were the classic success-oriented and failure-avoiding students: “Optimists” were high on success orientation and low on failure avoidance (cf. performance-approach oriented students: Nicholls, 1984; Elliot and Harackiewicz, 1996); “Self-protectors” were high on failure avoidance but low on success orientation (cf. performance-avoidant students: Nicholls, 1984; Elliot and Harackiewicz, 1996). The two other groups were “Failure acceptors” (low on both dimensions) and “Overstrivers” (high on both). Failure acceptors were indifferent to school achievement, having given up their efforts to avoid the implications of failure (Covington and Omelich, 1985). Overstrivers reflected an intense desire both to succeed and avoid failure. They perceived themselves as capable but feared that they may not be as worthy as their achievements indicated, exhibiting a hybrid quality of hope and fear (Covington and Omelich, 1991). Although a successful strategy for achievement in the short-term, in the long run Covington proposed their success would become an “intolerable burden” (1992, p. 89) because nobody can live up to perfection and avoid failure forever. Although anxiety may arouse overstrivers’ abilities and efforts, Covington (1992) highlighted the risk to their wellbeing as a core issue that should be of concern to researchers. It is these ‘negative’ motivations that students can associate with studying mathematics and science, and a range of achievement-related as well as psychological wellbeing outcomes that the current study adds to previous literature using a typological approach. Regardless of the theoretical approach taken, it appears that when negatively valenced constructs are included, an asynchronous type emerges, with resemblance to Covington and Omelich’s (1991) originally proposed overstrivers in terms of maladaptive outcomes. In this section we review studies that used a typological approach outside of EVT and included both positively and negatively valenced motivational constructs.

#### Achievement Goal Theory

Originally, achievement goal theory stipulated a dichotomy of goal orientations: mastery/task (striving for competence) or performance/ego (to demonstrate competence relative to others; Dweck, 1986; Ames, 1992). Performance goals were later divided into performance-avoidance (to avoid showing lack of competence) and performance-approach (to demonstrate relative competence) with evidence that only performance-avoidance had clear detrimental effects (Barron and Harackiewicz, 2001), and performance-approach may be adaptively paired with mastery goals within the “multiple goals framework” (e.g., Pintrich, 2000). There have been a number of person-oriented studies using achievement goal theory in domain-general, rather than STEM-specific ways (e.g., Tuominen-Soini et al., 2011, 2012). Among Finnish secondary school students, four achievement goal orientation profiles have been consistently found: (a) indifferent: those with scores close to the mean on all achievement goal orientations, therefore not displaying a tendency toward any particular orientation; (b) success-oriented: those with high levels of mastery and performance-related orientations; (c) mastery-oriented: having high mastery orientation and relatively low scores on all other orientations; and (d) avoidance-oriented: students low on mastery orientation who aim to minimise effort. In both those studies, aggregated single value measures at a domain-general level (i.e., school value) were included as a criterion. Mastery-oriented students had highest school value, followed by success-oriented students, with the other groups (avoidance-oriented and indifferent) lowest. Mastery-oriented students reported lowest feelings of inadequacy (other types did not differ from each other). Mastery- and success-oriented students had higher academic achievement; success-oriented and indifferent students had higher fear of failure (Tuominen-Soini et al., 2011). Success-oriented and indifferent students suffered greater exhaustion than other types; mastery- and avoidance-oriented students did not differ on exhaustion (Tuominen-Soini et al., 2012). Higher psychological wellbeing among the mastery- than success-oriented students, points to the negative effects of concerns regarding one’s competence, as theorised by Covington (1992). The success-oriented group resembled a profile identified in an early study by Roeser et al. (2002) with high motivation and achievement, and at the same time, emotional distress. While we do not measure students’ personal goal orientations in the current study, we do measure the perceived goal orientations of their learning environments (to what extent the teacher promotes a mastery or performance-oriented classroom).

#### Burnout and Engagement

There is a line of student burnout and engagement literature that focuses on the adverse effects of school-related stress and anxiety. A number of studies have identified profiles of students combining school engagement and burnout at a domain-general level (Tuominen-Soini and Salmela-Aro, 2014; Salmela-Aro and Read, 2017). The latter study found four groups; “engaged”, “engaged-exhausted”, “burned-out”, and “cynical” (disengaged but lower on burnout dimensions than “burned-out”). Engaged students reported the highest school value, GPA and psychological wellbeing. Engaged-exhausted had the second highest level of school value and GPA but more depressive symptoms, lower self-esteem and greater preoccupation with possible failure than engaged and cynical students. Cynical and burned-out students scored lowest on both school value and GPA; their distinguishing feature was their general psychological wellbeing – burned-out students suffered the most depressive symptoms and lowest self-esteem; cynical students had higher psychological wellbeing than burned-out and engaged-exhausted students. Three profiles of Finnish lower-secondary school students were also identified based on burnout together with academic-general values (beliefs about the importance of school) and self-regulation variables (effort and preparation; Virtanen et al., 2018): “high-engagement/low-burnout” (a positive type), “low-engagement/high-burnout” (a negative type), and “average-engagement/average-burnout” (an asynchronous type). The burnout and engagement literature shows, at a domain-general level, that combining positively and negatively valenced constructs results in synchronous as well as asynchronous profiles, which link to academic outcomes. However, researchers are yet to examine such links in a STEM-specific way.

#### Self-Regulation/Coping and Psychological Wellbeing

A combined focus on motivation, self-regulation and psychological wellbeing identified four profiles of Australian undergraduate students based on a combination of adaptive and maladaptive motivational constructs (self-belief, valuing of education, learning focus and failure avoidance), psychological wellbeing (anxiety) and self-regulation variables (task management, persistence and planning; Elphinstone and Tinker, 2017). As well as two groups characterised by high/low engagement and study skills, respectively, they identified two groups with moderate engagement – one accompanied by high maladaptive constructs (anxiety, failure avoidance, and uncertain control) and the other low. Thus, including negatively valenced constructs was important in distinguishing students having otherwise similar levels of motivation and engagement.

In an influential large-scale German study among health professionals (Schaarschmidt and Fischer, 1997), four types (clustered on 11 self-reported coping dimensions) were linked to achievement striving and psychological wellbeing. “Good psychological health” and “Sparing” exhibited positive psychological health, but differed in their professional commitment and work efforts; “Excessively ambitious” and “Burnout” were both risk types for poor psychological health, but the former showed excessive commitment to work (resembling overstrivers, although grounded in different measures and theory), whereas the “Burnout” were exhausted with reduced commitment to work. There appear to be similarities in patterns of profiles across different theoretical approaches and contexts (both country settings and level of schooling/workforce). It will be important to see how STEM-specific expectancies and values combine and relate to achievement striving, career aspirations, psychological wellbeing, gender, prior achievement, and experienced learning environments, among a sample of youth not yet self-selected into their STEM studies.

### Potential Outcomes: Achievement Striving and Career Aspirations

Achievement striving, career aspirations and dimensions of psychological wellbeing were expected to relate to expectancy-value motivational profiles. Some of the reviewed studies linked motivational profiles to career aspirations (Viljaranta et al., 2009; Chow et al., 2012; Chittum and Jones, 2017; Guo et al., 2018; Linnenbrink-Garcia et al., 2018) or achievement striving (Chittum and Jones, 2017; Ng, 2018) as criterion variables. Key findings, from studies which examined profiles within STEM domains together with STEM-related outcomes, linked profiles to aimed marks in a study framed by achievement goal theory (Ng, 2018), efforts exerted and STEM-related career aspirations in a study located within the expectancy-value framework (Chittum and Jones, 2017), and STEM career aspirations in a study drawing on both theories (Linnenbrink-Garcia et al., 2018).

In brief, “avoidant” Hong Kong secondary school students (high performance-avoidance, low mastery, and performance-approach) aimed for lowest grades, followed by “performance-anxious” (high performance-approach and avoidance); “all-goal” and “motivated” (relatively high mastery, performance-approach, and pro-social goals) students demonstrated similar high grade aspirations (Ng, 2018). United States fifth to seventh graders in a high interest/expectancy/utility value profile reported highest efforts and career aspirations in science, whereas a low interest/utility value profile reported the lowest, despite high success expectancies (Chittum and Jones, 2017). A United States college student “high intrinsic motivation and confidence” profile identified highest intentions for a science research career, while an “average” profile had the lowest intentions (Linnenbrink-Garcia et al., 2018).

Wellbeing-related factors were included as criterion variables in some of the already reviewed motivation profile studies (e.g., negative affect in mathematics class, Conley, 2012; exhaustion and feelings of inadequacy, Tuominen-Soini et al., 2012). There have been growing efforts to integrate psychological wellbeing and achievement motivation-related constructs, although not yet in a STEM-specific way.

### Potential Antecedents

We included the role of gender, prior achievement background and experienced learning environments as key potential antecedents.

#### Achievement Background and Gender

Despite equivalent abilities, a large literature has documented higher mathematics ability-related beliefs for boys versus girls (Eccles et al., 1983, 1984; Stevenson and Newman, 1986; Else-Quest et al., 2010), and girls’ lower beliefs in their capabilities for mathematical activities than boys’ are well-established with an effect size of *d* = 0.16 in Hyde’s (2005) meta-analysis. On the “values” side of EVT, adolescent girls and boys report similar beliefs about the utility/importance value of mathematics in Australian (Watt, 2004), United States and Canadian samples (Watt et al., 2012). However, boys consistently report higher interest in mathematics than girls (Updegraff et al., 1996; Watt, 2004; Frenzel et al., 2010), including in the PISA (Programme of International Student Assessment; Organisation for Economic Co-operation and Development [OECD], 2004) results, which showed higher mathematics interest and enjoyment for 15-year old boys than girls across all 41 participating countries. Less attention has been given to gender differences in *negative* motivational factors. An exception is mathematics anxiety, with an effect size showing worse anxiety for girls, *d* = −0.15 (Hyde, 2005), having obvious relevance to psychological or emotional cost. On this basis, more girls are expected to be in mathematics types showing low perceived talent and interest, as well as high psychological cost, but no hypothesis is advanced regarding gender composition of science profiles where there has not been the same volume of systematic study of gender differences, and where the domain (‘science’) encompasses a range of disciplines that may mask nuanced effects.

#### Learning Environments

Achievement goal theory (AGT) offers a framework within which to analyse students’ motivational learning environments as potential antecedents to motivational profiles. Mastery-oriented classrooms (focused on learning and understanding) have generally been found to predict higher levels of interest (Midgley et al., 2001; Harackiewicz et al., 2008; Carmichael et al., 2017), whereas performance-oriented classrooms (focused on competition and grades) tend to be unassociated (Harackiewicz et al., 2008; Pantziara and Philippou, 2015; Carmichael et al., 2017) or even undermine learning (Crouzevialle and Butera, 2013). Mastery and performance-oriented learning environments have been linked to students’ mathematical task values and career aspirations (Lazarides and Watt, 2015). There is large variation in students’ perceptions of the same classroom environment (Wolters, 2004; Spearman and Watt, 2013), pointing to the importance of factors which filter and frame students’ interpretations of their learning experiences, such as gender and motivational type, possibly fuelled by stereotypes that girls are not as naturally gifted at mathematics as boys. For example, Covington (1984) anticipated competitive learning environments to exacerbate stressors for overstrivers. We expected that experienced mastery environments would predominate among positive motivational types, and performance-oriented environments among a hybrid type characterised by high positive and negative cost motivations.

### The Present Study

Our core aims were, first, to validate the new adolescent multidimensional cost measure in the context of the set of expectancy-value constructs within each of mathematics and science, and present a rich nomological network of associations with demographics, achievement background, experienced learning environments, achievement striving, STEM-related career aspirations and dimensions of psychological wellbeing. Our second aim was to discern theoretically coherent profiles, explicitly grounded in the Eccles et al. (1983; Eccles, 2005, 2009) expectancy-value model, to link with potential antecedents and outcomes, and to assess the degree of domain specificity versus generality.

Using established measures of perceived talent, intrinsic and utility values, and a new multidimensional measure for seldom-researched “costs”, latent profile analysis (LPA) educed expectancy-value profiles among grade 10 students in each of mathematics and science. This person-centred approach allowed consideration of how motivational dimensions combined among different types of students to offer a nuanced understanding of the features and dynamics associated with particular profiles (Lawson and Lawson, 2013). Gender, experienced classroom learning environment and achievement influences were compared; and potential consequences for achievement striving, STEM-related career plans, and psychological wellbeing. It was hypothesised that profiles would be related but also distinct across mathematics/science, differentiated along positive/negative dimensions of achievement striving and psychological wellbeing.

Based on the preceding review, we advanced the following hypotheses:

(1) We expected to identify a positive profile (high perceived talent, intrinsic and utility values, low costs) with adaptive outcomes.
(2) We hypothesised that we would find an asynchronous type (high on all) with detrimental consequences for psychological wellbeing, although not for achievement striving or career aspirations.
(3) Finally, because the importance of mathematics and science are especially emphasised during school, we speculated that it may be unlikely that we identify a group low on all factors. Indeed, the studies by Lazarides et al. (2016a, b) detected a group for whom mathematics utility value was pronounced despite their low intrinsic value and self-concept. With cost factors simultaneously included, we speculated that the inconsistency between high utility value versus own low interests and self-beliefs may coincide with strain manifested in costs, leading to a mixed profile (high utility value, low perceived talent and intrinsic value, and potentially elevated costs) with lower achievement striving and STEM career aspirations, but less effect on psychological wellbeing.
(4) Experienced learning environments were expected to relate to motivational types, with performance climate more strongly experienced by an asynchronous type, mastery by the positive type, and no hypothesis was advanced for the third speculated type.

## Materials and Methods

### Sample

Data were from the Study of Transitions and Education Pathways^1^ (STEPS; Watt, 2004). Participants were grade 10 students (*N* = 1,172; 702 girls and 470 boys), before students have the option to opt out of mathematics and/or science for their final grades 11 and 12 of secondary schooling, recruited from 9 metropolitan/suburban schools in 2012/2013 from Melbourne and Sydney, Australia. Response rates ranged from 36 to 96% across schools (*Mdn* = 78%). Three schools were academically selective (i.e., students pass an achievement test to be able to enrol). School ICSEA (Index of Community Socio-Educational Advantage) scores ranged from 957 to 1,187 (*Mdn* = 1,128). Higher ICSEA scores indicate a higher level of educational advantage of students who attend that school, set at a national average value of 1,000. Students reported their parents’ highest level of education from 1 (did not complete high school), 2 (completed high school), 3 (completed TAFE training [Technical And Further Education colleges in Australia]), 4 (completed a university degree). Table 1 depicts the sample size for each cohort, percentage response rate, proportion of girls, school ICSEA and selective schools. Mean age was 15.79 in years (*SD* = 0.50). The sample was predominantly of English-speaking background (*n* = 754), with Chinese as the next frequent home language (*n* = 121), followed by Vietnamese (*n* = 92), Sinhalese (*n* = 23), and Korean (*n* = 18); other home language frequencies were 10 or fewer. Home language background was coded 1 (English) or 0 (other language).

**TABLE 1:** Sample response rates and distribution per year by school, cohort and gender (*N* = 1,172).

	**2012**					**2013**					
School	1^a^	2	3	4	5	4	5	6^a^	7^a^	8	9
Cohort	1	2	3	4	5	6	7	8	9	10	11
*N*	50	74	186	54	74	70	78	178	182	159	67
% response	36	81	76	51	96	78	88	77	91	56	83
% girls	100	45	47	100	0	100	0	100	40	45	100
ICSEA^b^	1187	1157	957	1051	1078	1051	1078	1128	1123	1184	1163

**^*a*^Selective schools.^*b*^ICSEA (Index of Community Socio-Educational Advantage) is a scale set at an average of 1,000 which allows for comparisons among schools with similar students. The higher the ICSEA, the higher the level of educational advantage of students who go to that school; the lower the ICSEA, the lower the level of students’ educational advantage. For comparability the 2012 scores were used for all schools*.*

### Procedure

Principals of participating schools were sent an invitation letter by the first author outlining the study design and rationale, including required university, school system and departmental ethical approvals, followed up with a telephone call one week later. Meetings with each principal and selected senior school staff followed, following which the included nine schools agreed they were willing to facilitate the study. Invitation letters, explanatory statements and participation consent forms were distributed by school year coordinators to students, who were requested to return them to indicate their own and parents’ consent to participate or otherwise. Surveys were administered by the first author and trained research assistants to participating students during negotiated class time in approximately the third month of the school year and took approximately 30 minutes to complete.

### Measures

The second Study of Transitions and Education Pathways was designed to tap a range of students’ motivational constructs and aspirations relevant to mathematics and science, building on previous work in the expectancy-value model (Watt, 2004), achievement background and aspirations, learning environments and psychological wellbeing.

#### Expectancies and Values

Motivational constructs were assessed in relation to each of mathematics and science using Eccles and colleagues’ expectancy-value measures (Eccles and Wigfield, 1995), with contextualising modifications for the Australian sample. Table 2 presents a complete items list and acceptable Cronbach’s alpha measures of internal consistency. Students’ perceptions of talent, intrinsic and utility task values were assessed by items adapted to the Australian setting (see Watt, 2002, 2004) on 7-point Likert scales ranging from 1 (not at all) to 7 (extremely). Perceived talent was tapped by four items, e.g., “Compared with other students in your class, how talented do you consider yourself to be at maths[/science]?”. Intrinsic value was measured by three items, e.g., “How much do you like maths[/science], compared with your other subjects at school?”. Utility value was measured by three items, e.g., “How useful do you believe maths[/science] is?”

**TABLE 2:** Expectancy-value constructs and items.

**Construct (α) (math/science)**	**Item**
*Perceived talent* (0.91/0.93): 1 = not at all, 7 = extremely	
	Compared with other students in your class, how talented do you consider yourself to be at maths/science?
	Compared with other students in your Year, how talented do you consider yourself to be at maths/science?
	Compared with your friends, how talented do you consider yourself to be at maths/science?
	Compared with other subjects at school, how talented do you consider yourself to be at maths/science?
*Intrinsic value* (0.95/0.94): 1 = not at all, 7 = extremely	
	How much do you like maths/science compared with your other subjects at school?
	How interesting do you find maths/science?
	How enjoyable do you find maths/science?
*Utility value* (0.89/0.89): 1 = not at all, 7 = extremely	
	How useful do you believe maths/science is?
	How useful do you think maths/science is in the everyday world?
	How useful do you think mathematical/scientific skills are in the workplace?
*Cost-effort* (0.77/0.86): 1 = strongly disagree, 7 = strongly agree	
	When I think about the hard work needed to get through in maths/science, I am not sure that it is going to be worth it in the end.
	Considering what I want to do with my life, studying maths/science is just not worth the effort.
	Achieving in maths/science sounds like it really requires more effort than I’m willing to put into it.
*Cost-psychological* (0.85/0.86): 1 = strongly disagree, 7 = strongly agree	
	It frightens me that maths/science courses are harder that other courses.
	I’m concerned that I won’t be able to handle the stress that goes along with studying maths/science.
*Cost-social* (0.81/0.89): 1 = strongly disagree, 7 = strongly agree	
	I’m concerned that working hard in maths/science classes might mean I lose some of my close friends.
	I worry about losing some valuable friendships if I’m studying maths/science and my friends are not.

A new multidimensional costs measure was developed for adolescents, adapted from Perez et al.’s (2014) college-level scale, tapping Effort cost (3 items, e.g., “Achieving in maths[/science] sounds like it really requires more effort than I’m willing to put into it”), Psychological cost (2 items, e.g., “I’m concerned that I won’t be able to handle the stress that goes along with studying maths[/science]”) and Social cost (2 items, e.g., “I’m concerned that working hard in maths[/science] classes might mean I lose some of my close friends”). The Social cost factor was renamed from “opportunity cost” referred to by Perez et al. (2014) to better reflect these items’ content. All costs were rated from 1 = strongly disagree, to 7 = strongly agree. Validation of this new measure was established as part of the outlined confirmatory factor analyses that follow.

#### Achievement Background

Achievement background in mathematics was assessed by students’ performance on the grade 9 national numeracy examination ‘NAPLAN’ (National Assessment Program – Literacy and Numeracy) from Band 5 (lowest) to Band 10 (highest). In science, for which no common assessment is undertaken, students self-reported their ‘usual’ grade 9 score selected from 0 (<50%), 1 (50–54%), 2 (55–59%), 3 (60–64%), 4 (65–69%), 5 (70–74%), 6 (75–79%), 7 (80–84%), 8 (85–89%), 9 (90–94%), 10 (95–100%).

#### Perceived Classroom Learning Environment

Students’ perceived mathematics/science classroom learning environments were assessed for performance-approach (e.g., “Our maths[/science] teacher points out those students who get good grades as an example to all of us”) and mastery goal orientations (e.g., “Our maths[/science] teacher really wants us to enjoy learning new things”). All items were measured by 7-point Likert scales ranging from 1 (not at all) to 7 (extremely) using items from the Patterns of Adaptive Learning Scales (PALS; Midgley et al., 2000). For mastery orientation, three out of five items from PALS were administered in the interest of total survey length (αs = 0.85/0.88 for mathematics/science, respectively); for performance-approach, all three items were used (αs = 0.80/0.87).

#### Achievement Striving

Aimed marks were measured by the question “What mark do you (realistically) aim for in Year 10 maths[/science] exams?”, selected from 0 (<50%), 1 (50–54%), 2 (55–59%), 3 (60–64%), 4 (65–69%), 5 (70–74%), 6 (75–79%), 7 (80–84%), 8 (85–89%), 9 (90–94%), 10 (95–100%). Effort exertion was tapped by two items (e.g., “How much effort do you put into maths[/science]?”) rated from 1 = not at all, to 7 = extremely (αs = 0.77/0.86).

#### STEM Career Aspirations

Mathematics/science-related career aspirations were subjectively assessed by the question “How much would you like to have a maths[/science]-related career?”, rated from 1 (not at all) to 7 (extremely). As well, students responded to an open-ended question: “What career are you mainly considering for your future?”. Each career plan was subsequently objectively coded for the extent of mathematics/physics/chemistry/biology/ engineering knowledge required for that occupation, using the United States Department of Labor O^*^NET 2016 (National Center for O^*^NET Development, 2016), based on data from workers and occupation experts. This yielded a continuous score for each STEM dimension (from 0 to 100) for knowledge required per occupation. For example, for physiotherapists: mathematics = 38, physics = 43, chemistry = 31, biology = 74, engineering = 17. If students listed more than one occupation, the one that they listed first was coded. Occupational data could be coded for 796 of the 824 participants who nominated a career plan.

#### Psychological Wellbeing

Psychological wellbeing was measured by the DASS_21_ (Depression Anxiety Stress Scales; Lovibond and Lovibond, 1995), tapping 3 factors measured by 21 statements rated in relation to the past week (0: did not apply to me at all; 1: applied to me to some degree, or some of the time; 2: applied to me to a considerable degree, or a good part of the time; 3: applied to me very much, or most of the time). The DASS has been used in a range of previous studies to assess psychological wellbeing (e.g., Chu and Richdale, 2009; Asante, 2012; Giallo et al., 2013; Larcombe et al., 2013; Sharp et al., 2016). Sample items were “I felt that I had nothing to look forward to” for Depression, “I felt I was close to panic” for Anxiety, and “I tended to over-react to situations” for Stress (see Table 3).

**TABLE 3:** Depression, anxiety, stress (DASS) constructs and items.

*Depression* (α = 0.88)	
	I found it hard to wind down.
	I couldn’t seem to experience any positive feeling at all.
	I found it difficult to work up the initiative to do things.
	I felt that I had nothing to look forward to.
	I felt down-hearted and blue.
	I was unable to become enthusiastic about anything.
	I felt I wasn’t worth much as a person.
	I felt that life was meaningless.
*Anxiety* (α = 0.84)	
	I was aware of dryness of my mouth.
	I experienced breathing difficulty (e.g., excessively rapid breathing, breathlessness in the absence of physical exertion).
	I experienced trembling (e.g., in the hands).
	I was worried about situations in which I might panic and make a fool of myself.
	I felt I was close to panic.
	I was aware of the action of my heart in the absence of physical exertion (e.g., sense of heart rate increase, heart missing a beat).
	I felt scared without any good reason.
*Stress* (α = 0.85)	
	I felt that I was rather touchy.
	I tended to over-react to situations.
	I felt that I was using a lot of nervous energy.
	I found myself getting agitated.
	I found it difficult to relax.
	I was intolerant of anything that kept me from getting on with what I was doing.

**0 = Did not apply to me at all; 1 = applied to me to some degree, or some of the time; 2 = applied to me to a considerable degree, or a good part of time; 3 = applied to me very much, or most of the time (“over the past week”).**

Gender was coded 0 for boys, 1 for girls.

### Analyses

Confirmatory factor analysis for each of the mathematics and science latent motivation constructs (perceived talent, intrinsic and utility values, together with the new items tapping effort/psychological/social costs), and psychological wellbeing constructs (depression, anxiety, and stress) were specified, with items as indicators for only their assigned latent constructs, no cross-loadings or error covariances, using listwise deletion for low missing data initially. Fit adequacy was assessed following Hu and Bentler’s (1999) two-index strategy, according to the comparative fit index (CFI ≥ 0.95) and standardised root mean square residual (SRMR ≤ 0.09), as well as the root mean square error of approximation (RMSEA < 0.06) and Tucker-Lewis Index (TLI close to 0.95). Data showed an acceptable fit for each of the mathematics (χ^2^ = 691.31, df = 131, CFI = 0.96, TLI = 0.95, RMSEA = 0.06, SRMR = 0.04) and science models (χ^2^ = 764.77, df = 131, CFI = 0.96, TLI = 0.95, RMSEA = 0.07, SRMR = 0.04), with details reported in Table 4. The fit was marginal for the psychological wellbeing model (χ^2^ = 1283.72, df = 186, CFI = 0.91, TLI = 0.89, RMSEA = 0.08, SRMR = 0.05), subsequently improved through correlating measurement errors for Depression items “I felt I wasn’t worth much as a person” (DASS17) and “I felt that life was meaningless” (DASS21) (TD = 0.40 standardised estimate; revised model fit χ^2^ = 1140.29, df = 185, CFI = 0.92, TLI = 0.91, RMSEA = 0.07, SRMR = 0.05); see Table 5.

**TABLE 4:** Confirmatory factor analysis factor loadings (LX) and measurement errors (TD) for mathematics and science expectancy-value constructs and Cronbach alpha reliabilities.

	**Subscale α**	**Item**	**LX**	**TD**
Talent	0.91/0.93	TAL1^*^	0.813/0.892	0.627/0.361
		TAL2	0.847/0.902	0.698/0.411
		TAL3	0.832/0.899	0.674/0.405
		TAL4	0.879/0.858	0.576/0.583
Intrinsic	0.95/0.94	INT1^*^	0.929/0.917	0.364/0.418
		INT2	0.942/0.911	0.298/0.441
		INT3	0.930/0.933	0.369/0.347
Utility	0.89/0.89	UTIL1^*^	0.847/0.888	0.633/0.503
		UTIL2	0.895/0.855	0.457/0.687
		UTIL3	0.849/0.817	0.518/0.859
Effort	0.77/0.86	EFF1^*^	0.907/0.950	0.325/0.186
		EFF2	0.961/0.940	0.145/0.227
Cost–effort	0.77/0.86	EFFC1^*^	0.751/0.837	1.116/0.816
		EFFC2	0.776/0.828	0.971/0.929
		EFFC3	0.682/0.786	1.488/1.111
Cost–psychological	0.85/0.86	PSYC1^*^	0.866/0.900	0.830/0.626
		PSYC2	0.850/0.832	0.947/1.011
Cost–social	0.81/0.89	SOCC1^*^	0.854/0.926	0.507/0.264
		SOCC2	0.811/0.887	0.635/0.391

^*^Item set as reference per subscale. Coefficients are reported adjacent for mathematics (left side) and science models (right side).

**TABLE 5:** Confirmatory factor analysis factor loadings (LX) and measurement errors (TD) for DASS constructs and Cronbach alpha reliabilities.

	**Subscale α**	**Item**	**LX**	**TD**
Depression	0.88	DASS1^*^	0.492	0.694
		DASS3	0.727	0.401
		DASS5	0.607	0.658
		DASS10	0.746	0.413
		DASS13	0.810	0.299
		DASS16	0.778	0.326
		DASS17	0.759	0.362
		DASS21	0.704	0.461
Anxiety	0.84	DASS2^*^	0.479	0.774
		DASS4	0.615	0.473
		DASS7	0.689	0.397
		DASS9	0.655	0.629
		DASS15	0.777	0.306
		DASS19	0.683	0.417
		DASS20	0.712	0.379
Stress	0.85	DASS6^*^	0.658	0.587
		DASS8	0.741	0.424
		DASS11	0.750	0.412
		DASS12	0.728	0.475
		DASS14	0.669	0.493
		DASS18	0.670	0.459

^*^Item set as reference per subscale. Standardised error covariance between DASS17 and DASS18 TD = 0.397.

Latent profile analysis (LPA) educed profiles within each of mathematics and science, among the 1,172 Australian grade 10 students’ perceived talent, intrinsic and utility values, and effort/psychological/social costs. The intraclass correlation (ICC) reveals the extent to which individual responses are attributable, in this case, to mathematics/science classroom membership (calculated by dividing the between-cluster variance by the between- plus within-cluster variance; Raudenbush and Bryk, 2002). ICC ≥ 0.05 may provide evidence of a classroom membership effect (LeBreton and Senter, 2008). A more definitive indicator is the design effect (*deff*), a function of the ICC and average cluster size [approximately equal to 1 + (average cluster size − 1) × ICC; Kish, 1965]. *deff* ≥ 2 indicates that the clustered structure should be considered to avoid biassed estimates of standard errors (Maas and Hox, 2004). *deff* values on the clustering variables were well below 2.0 (see Table 6), except for students’ perceived talent in each of mathematics/science, presumably due to achievement-streamed classes in secondary schooling. We therefore accounted for non-independence of observations^2^ of students within each of their 74 mathematics and 74 science classes (see Asparouhov, 2005, 2006; Maas and Hox, 2005) by employing the robust maximum likelihood estimator and design-based correction for standard errors and chi-square test of model fit available within *Mplus* version 8.1 (Type = Mixture Complex). Mean numbers of student respondents across classes were 15.78 in mathematics (*SD* = 6.60) and 15.80 in science (*SD* = 7.64). Cases that had missing values for all variables (*n* = 4 in mathematics, 15 in science) or for the classroom variable (*n* = 4 in mathematics, 3 in science) were excluded from each analysis and FIML was employed to address the low remaining missing data.

**TABLE 6:** ICC and design effect for clustering variables.

	**Mathematics**		**Science**	
	**ICC**	***deff***	**ICC**	***deff***
Perc. talent	0.34	6.07	0.15	3.23
Intrinsic value	0.31	0.69	0.21	0.79
Utility value	0.12	0.88	0.19	0.81
Effort cost	0.14	0.86	0.17	0.83
Psych. cost	0.07	0.93	0.13	0.87
Social cost	0.08	0.92	0.06	0.94

**deff, design effect.**

A series of analyses compared one to five latent profile models whose fit indices are presented in Table 8. The optimal number of profiles was decided based on a range of widely used statistical criteria (Nylund et al., 2007), supported by substantive interpretability: the Akaike information criterion (AIC; Akaike, 1974), Bayesian information criterion (BIC; Schwarz, 1978), sample-size-adjusted Bayesian information criterion (aBIC), entropy, adjusted Lo–Mendell–Rubin Likelihood Ratio Test (LMR LRT), and Vuong–Lo–Mendell–Rubin Likelihood Ratio Test (VLMR LRT). The optimised solution is expected when values for the AIC/BIC/aBIC are lowest – or when the plotted curve begins to flatten (Masyn, 2013) – indicating that little further information would be gained through additional profiles; entropy >0.80 (Rost, 2006); and LMR/VLMR LRT *p-*values indicate when the *k*−1 class null model should be rejected in favour of *k* classes.

Subsequently, using SPSS (version 24) and listwise deletion for missing data, initial ANOVAs compared achievement backgrounds by profiles in mathematics and science, and potential dependency on selective schools in mathematics. Seven M/ANOVAs with Bonferroni protected *p*-values were then conducted comparing each of mathematics (with and without the NAPLAN achievement covariate) and science profiles, on each of (i) the motivation constructs from which profiles were educed (i.e., perceived talent, intrinsic and utility values, effort/psychological/social costs), (ii) achievement background, (iii) experienced learning environments (mastery and performance goal structures), (iv) aimed marks, (v) effort exerted and subjective mathematics/science career aspiration, (vi) objectively coded STEM career plans, and (vii) psychological wellbeing (depression, anxiety, stress). Chi-square tested associations of profiles with gender, language background, and mothers’/fathers’ level of education.

## Results

To address our first central aim, associations were examined among mathematics/science expectancy-value motivations including costs and potential antecedent (demographics, achievement background, and learning environment) and outcome factors (achievement striving, career aspirations, and psychological wellbeing). This yielded a rich nomological network for the new cost factors, including psychological wellbeing together with achievement-related outcomes. For our second aim, latent profiles of students were educed in each domain, cross-domain membership was examined, and potential antecedents and outcomes were compared.

### Associations Among Motivations, Potential Antecedents, Achievement Striving, Career Aspirations, and Psychological Wellbeing

Latent correlations among all latent and observed constructs (see Table 7) were obtained from a combined CFA including demographic factors (gender, English home language, mother and father highest level of education, selective school), mathematics and science achievement background (grade 9 NAPLAN band and usual science score, respectively), expectancy-value constructs in each of mathematics and science (perceived talent, intrinsic and utility values, effort/psychological/ social costs), mathematics and science class mastery and performance climates, aimed marks and effort exerted, mathematics/science-related career aspirations, and psychological wellbeing (depression, anxiety, and stress). In this model, items were specified only as indicators of their respective theorised latent constructs, measurement errors were covaried between the same items pertaining to mathematics/science parallel constructs (due to their parallel wording), the measurement error covariance between depression items was retained (DASS 17, 21), and all latent correlations between constructs were estimated using full information maximum likelihood to account for missing data. The data fitted the model acceptably, χ^2^ = 6481.79, df = 2978, CFI = 0.95, TLI = 0.93, RMSEA = 0.03. Relationships between core study constructs are highlighted below (Table 7 shows all relationships for completeness).

**TABLE 7:** Latent correlations between study constructs.

	**1**	**2**	**3**	**4**	**5**	**6**	**7**	**8**	**9**	**10**	**11**	**12**	**13**	**14**	**15**	**16**	**17**	**18**	**19**	**20**	**21**	**22**	**23**	**24**	**25**	**26**	**27**	**28**	**29**	**30**	**31**	**32**	**33**	**34**	**35**	**36**	**37**
(1) Gender	–																																				
(2) Language	–0.04	–																																			
(3) MoEd level	–0.02	0.15^∗∗^	–																																		
(4) FaEd level	0.03	0.07^†^	0.54^∗∗^	–																																	
(5) Selective school	0.21^∗∗^	–0.16^∗∗^	0.14^∗∗^	0.14^∗∗^	–																																
(6) M NAPLAN ach.	0.04	–0.12^∗∗^	0.22^∗∗^	0.22^∗∗^	0.47^∗∗^	–																															
(7) S ‘usual’ ach.	0.14^∗∗^	–0.11^∗∗^	0.14^∗∗^	0.12^∗∗^	0.43^∗∗^	0.54^∗∗^	–																														
(8) M talent	–0.18^∗∗^	–0.13^∗∗^	0.08^†^	0.13	0.01^*^	0.45^∗∗^	0.34^∗∗^	–																													
(9) M intrinsic	–0.17^∗∗^	–0.19	0.07^†^	0.12^∗∗^	0.25^∗∗^	0.39^∗∗^	0.32^∗∗^	0.73^∗∗^	–																												
(10) M utility	–0.15^∗∗^	−0.08^*^	0.05	0.08^†^	0.14^∗∗^	0.16^∗∗^	0.24^∗∗^	0.44^∗∗^	0.57^∗∗^	–																											
(11) M cost-eff	0.02	0.03	−0.08^†^	−0.09^*^	–0.27^∗∗^	–0.31^∗∗^	–0.36^∗∗^	–0.46^∗∗^	–0.56^∗∗^	–0.54^∗∗^	–																										
(12) M cost-psy	0.09^*^	0.06	−0.08^†^	−0.09^*^	–0.16^∗∗^	–0.23^∗∗^	–0.18^∗∗^	–0.31^∗∗^	–0.34^∗∗^	–0.13^∗∗^	0.52^∗∗^	–																									
(13) M cost-soc	–0.20^∗∗^	–0.05	−0.08^†^	−0.07^†^	–0.17^∗∗^	–0.13^∗∗^	–0.16^∗∗^	0.03	–0.05	−0.08^†^	0.40^∗∗^	0.44^∗∗^	–																								
(14) M mastery	–0.17^∗∗^	–0.02	–0.02	0.04	0.04	0.01^*^	0.05	0.22^∗∗^	0.33^∗∗^	0.29^∗∗^	–0.18^∗∗^	–0.04	–0.05	–																							
(15) M perf.	–0.30^∗∗^	0.17^∗∗^	0.05	0.03	–0.30^∗∗^	–0.06	–0.19^∗∗^	0.01^*^	0.05	0.04	0.08^†^	0.03	0.21^∗∗^	0.21^∗∗^	–																						
(16) M aimed%	0.02	–0.25^∗∗^	0.11^∗∗^	0.15^∗∗^	0.35^∗∗^	0.52^∗∗^	0.55^∗∗^	0.60^∗∗^	0.56^∗∗^	0.31^∗∗^	–0.42^∗∗^	–0.23^∗∗^	–0.06	0.12^∗∗^	−0.09^*^	–																					
(17) M effort	–0.06	–0.04	0.01^*^	0.07	0.09^*^	0.17^∗∗^	0.23^∗∗^	0.47^∗∗^	0.55^∗∗^	0.37^∗∗^	–0.38^∗∗^	–0.11^∗∗^	–0.02	0.26^∗∗^	0.03	0.37^∗∗^	–																				
(18) S talent	–0.08	0.04	0.14^∗∗^	0.06^†^	0.06	0.30^∗∗^	0.55^∗∗^	0.44^∗∗^	0.28^∗∗^	0.26^∗∗^	–0.27^∗∗^	–0.15^∗∗^	–0.02	0.10^*^	0.04	0.27^∗∗^	0.25^∗∗^	–																			
(19) S intrinsic	0.00	−0.07^†^	0.01^*^	0.04	0.24^∗∗^	0.28^∗∗^	0.53^∗∗^	0.30^∗∗^	0.35^∗∗^	0.36^∗∗^	–0.35^∗∗^	–0.17^∗∗^	−0.10^*^	0.15^∗∗^	−0.10^*^	0.28^∗∗^	0.27^∗∗^	0.71^∗∗^	–																		
(20) S utility	0.06	–0.12^∗∗^	0.09	0.01^*^	0.30^∗∗^	0.33^∗∗^	0.48^∗∗^	0.33^∗∗^	0.38^∗∗^	0.42^∗∗^	–0.36^∗∗^	–0.16^∗∗^	–0.14^∗∗^	0.13^∗∗^	–0.14^∗∗^	0.37^∗∗^	0.27^∗∗^	0.52^∗∗^	0.75^∗∗^																		
(21) S cost-effort	–0.05	0.08^*^	−0.10^*^	−0.10^*^	–0.32^∗∗^	–0.31^∗∗^	–0.49^∗∗^	–0.23^∗∗^	–0.26^∗∗^	–0.31^∗∗^	0.54^∗∗^	0.32^∗∗^	0.30^∗∗^	−0.11^*^	0.16^∗∗^	–0.26^∗∗^	–0.22^∗∗^	–0.52^∗∗^	–0.66^∗∗^	–0.56^∗∗^	–																
(22) S cost-psych	0.15^∗∗^	–0.03	–0.14^∗∗^	−0.10^*^	−0.10^*^	–0.12^∗∗^	–0.23^∗∗^	–0.12^∗∗^	–0.11^∗∗^	−0.10^*^	0.27^∗∗^	0.55^∗∗^	0.30^∗∗^	−0.07^†^	0.03	–0.04	−0.07^*^	–0.33^∗∗^	–0.31^∗∗^	–0.14^∗∗^	0.58^∗∗^	–															
(23) S cost-social	–0.21^∗∗^	–0.04	−0.01^*^	−0.09^*^	–0.20^∗∗^	–0.14^∗∗^	–0.22^∗∗^	0.00	–0.03	−0.07^†^	0.30^∗∗^	0.26^∗∗^	0.71^∗∗^	–0.04	0.24^∗∗^	−0.07^†^	–0.04	−0.10^*^	–0.19^∗∗^	–0.23^∗∗^	0.45^∗∗^	0.43^∗∗^	–														
(24) S mastery	0.04	0.04	0.04	–0.01	0.11^∗∗^	0.07	0.18^∗∗^	0.10^*^	0.08^†^	0.15^∗∗^	–0.14^∗∗^	–0.05	–0.14^∗∗^	0.29^∗∗^	–0.02	0.03	0.13^∗∗^	0.27^∗∗^	0.43^∗∗^	0.32^∗∗^	–0.23^∗∗^	−0.09^*^	–0.13^∗∗^	–													
(25) S perf.	–0.30^∗∗^	0.10	–0.02	–0.03	–0.35^∗∗^	–0.15^∗∗^	–0.19^∗∗^	0.02	–0.03	–0.02	0.10^*^	0.07	0.22^∗∗^	0.08^†^	0.59^∗∗^	–0.18^∗∗^	−0.07^†^	0.09^*^	–0.05	−0.08^†^	0.17^∗∗^	0.05	0.30^∗∗^	0.10^*^	–												
(26) S aimed%	0.14^∗∗^	–0.13^∗∗^	0.14	0.12^∗∗^	0.33^∗∗^	0.50^∗∗^	0.77^∗∗^	0.36^∗∗^	0.32^∗∗^	0.24^∗∗^	–0.34^∗∗^	–0.15^∗∗^	–0.14^∗∗^	0.07^†^	–0.16^∗∗^	0.68^∗∗^	0.25^∗∗^	0.56^∗∗^	0.56^∗∗^	0.53^∗∗^	–0.47^∗∗^	–0.16^∗∗^	–0.18^∗∗^	0.21^∗∗^	–0.17^∗∗^	–											
(27) S effort	0.05	–0.03	0.09^*^	–0.01	0.18	0.20^∗∗^	0.43^∗∗^	0.25^∗∗^	0.26	0.26	–0.27^∗∗^	–0.07^∗∗^	–0.07	0.09^*^	−0.09^*^	0.24^∗∗^	0.46^∗∗^	0.59^∗∗^	0.71^∗∗^	0.58^∗∗^	–0.48^∗∗^	–0.13^∗∗^	–0.01	0.36^∗∗^	–0.01	0.48^∗∗^	–										
(28) M career	–0.19^∗∗^	–0.17^∗∗^	–0.01	0.02	0.13^∗∗^	0.26^∗∗^	0.24^∗∗^	0.57^∗∗^	0.64^∗∗^	0.51^∗∗^	–0.48^∗∗^	–0.23^∗∗^	–0.01	0.19^∗∗^	0.09^*^	0.40^∗∗^	0.33^∗∗^	0.23^∗∗^	0.24^∗∗^	0.29^∗∗^	–0.24^∗∗^	−0.08^†^	0.04	0.05	0.05	0.23^∗∗^	0.20^∗∗^	–									
(29) S career	0.03	–0.12^∗∗^	0.06^†^	0.04	0.32^∗∗^	0.32^∗∗^	0.50^∗∗^	0.33^∗∗^	0.36^∗∗^	0.36^∗∗^	–0.40^∗∗^	–0.14^∗∗^	−0.11^*^	0.14^∗∗^	−0.09^*^	0.37^∗∗^	0.25^∗∗^	0.55^∗∗^	0.71^∗∗^	0.67^∗∗^	–0.66^∗∗^	–0.21^∗∗^	–0.18^∗∗^	0.23^∗∗^	−0.07^†^	0.51^∗∗^	0.47^∗∗^	0.38^∗∗^	–								
(30) O^*^NET biology	0.18^∗∗^	–0.12^∗∗^	0.02	0.04	0.20^∗∗^	0.14^∗∗^	0.28^∗∗^	0.18^∗∗^	0.20^∗∗^	0.15^∗∗^	–0.23^∗∗^	−0.07^†^	−0.07^†^	0.02	−0.12^*^	0.19^∗∗^	0.16^∗∗^	0.28^∗∗^	0.39^∗∗^	0.38^∗∗^	–0.40^∗∗^	–0.04	−0.08^†^	0.08^†^	–0.12^∗∗^	0.28^∗∗^	0.28^∗∗^	0.17^∗∗^	0.56^∗∗^	–							
(31) O^*^NET chem.	0.01	–0.15^∗∗^	0.03	0.03	0.26^∗∗^	0.19^∗∗^	0.31^∗∗^	0.24^∗∗^	0.29^∗∗^	0.26^∗∗^	–0.32^∗∗^	–0.15^∗∗^	–0.05	0.09^†^	−0.11^*^	0.24^∗∗^	0.21^∗∗^	0.30^∗∗^	0.44^∗∗^	0.41^∗∗^	–0.45^∗∗^	−0.10^*^	–0.04	0.10^*^	−0.11^*^	0.28^∗∗^	0.30^∗∗^	0.29^∗∗^	0.58^∗∗^	0.80^∗∗^	–						
(32) O^*^NET physics	–0.25^∗∗^	–0.05^∗∗^	0.01	0.01	0.17^∗∗^	0.15^∗∗^	0.22^∗∗^	0.22^∗∗^	0.28^∗∗^	0.27^∗∗^	–0.30^∗∗^	–0.19^∗∗^	–0.01	0.14^∗∗^	0.03	0.20^∗∗^	0.19^∗∗^	0.21^∗∗^	0.31^∗∗^	0.27^∗∗^	–0.32^∗∗^	–0.17^∗∗^	0.01	0.07^†^	–0.02	0.18^∗∗^	0.19^∗∗^	0.34^∗∗^	0.38^∗∗^	0.31^∗∗^	0.56^∗∗^	–					
(33) O^*^NET math	–0.29^∗∗^	–0.13^∗∗^	0.03	–0.01	0.20^∗∗^	0.14^∗∗^	0.19^∗∗^	0.26^∗∗^	0.37^∗∗^	0.33^∗∗^	–0.37^∗∗^	–0.20^∗∗^	0.02	0.16^∗∗^	0.04	0.22^∗∗^	0.23^∗∗^	0.18^∗∗^	0.29^∗∗^	0.24^∗∗^	–0.27^∗∗^	–0.15^∗∗^	0.05	0.08^†^	–0.03	0.16^∗∗^	0.22^∗∗^	0.44^∗∗^	0.31^∗∗^	0.20^∗∗^	0.54^∗∗^	0.67^∗∗^	–				
(34) O^*^NET engineer	–0.34^∗∗^	–0.04	0.00	0.02	0.08	0.13^∗∗^	0.10^*^	0.13^∗∗^	0.17^∗∗^	0.22^∗∗^	–0.21^∗∗^	−0.11^*^	0.01	0.11^*^	0.07^†^	0.12^∗∗^	0.10^*^	0.05^†^	0.12^∗∗^	0.09^*^	–0.14^∗∗^	–0.15^∗∗^	0.02	0.03	0.02	0.07^†^	0.08^†^	0.27^∗∗^	0.11^∗∗^	–0.20^∗∗^	0.20^∗∗^	0.69^∗∗^	0.58^∗∗^	–			
(35) Depression	0.02	–0.02	−0.08^†^	0.00	–0.04	–0.12^∗∗^	–0.12^∗∗^	–0.11^∗∗^	–0.12^∗∗^	−0.10^†^	0.25^∗∗^	0.32^∗∗^	0.25^∗∗^	−0.09^†^	0.05	−0.07^†^	–0.16^∗∗^	–0.12^∗∗^	–0.12^∗∗^	–0.05	0.23^∗∗^	0.25^∗∗^	0.25^∗∗^	–0.12^∗∗^	0.09^*^	−0.09^*^	–0.12^∗∗^	–0.03	−0.10^*^	–0.04	–0.07	−0.09^†^	–0.12^∗∗^	–0.04	–		
(36) Anxiety	0.01	−0.07^†^	−0.10^*^	–0.01	−0.07^†^	–0.14^∗∗^	–0.13^∗∗^	−0.07^†^	–0.05	–0.01	0.20^∗∗^	0.32^∗∗^	0.28^∗∗^	–0.02	0.07	–0.06	–0.06	−0.08^†^	−0.07^†^	–0.02	0.18^∗∗^	0.26^∗∗^	0.26^∗∗^	−0.07^†^	0.12^∗∗^	−0.10^*^	–0.05	0.01	–0.08^∗∗^	–0.06	–0.06	–0.05	–0.05	0.02	0.81^∗∗^	–	
(37) Stress	0.11^∗∗^	–0.05	–0.06	0.02	0.00	−0.09^*^	–0.06	–0.05	–0.03	0.02	0.11^*^	0.34^∗∗^	0.21^∗∗^	–0.02	0.04	0.00	–0.02	−0.07^†^	–0.06	0.03	0.14^∗∗^	0.28^∗∗^	0.18^∗∗^	–0.07	0.07^†^	–0.03	–0.05	–0.01	–0.05	–0.01	–0.02	–0.06	–0.07	–0.04	0.85^∗∗^	0.93^∗∗^	–

Gender coded 1 = girls; Language coded 1 = English; Selective school coded 1 = Selective. ‘M’ denotes mathematics, ‘S’ science factors. ${}^{†}$p < 0.05, ^*^p < 0.01, ^∗∗^p < 0.001.

#### Student Gender

In mathematics, gender was unrelated to NAPLAN achievement background, achievement striving in the form of aimed marks and effort exerted. Yet, gender was weakly negatively associated with mathematical motivations of perceived talent, intrinsic and utility values, and social cost (boys higher), and positively with psychological cost (girls higher). Gender negatively related to mathematics mastery and performance-oriented classroom learning environments. Similarly, for science, gender was weakly positively associated with aimed marks, but not effort exertion. Gender was positively associated with social cost and negatively associated with psychological cost, but unassociated with any other science motivation. Gender negatively related to performance-oriented science learning environments (unrelated to mastery), and subjectively reported mathematical career aspirations (but not science career). For objectively scored STEM-related career plans according to O^*^NET, biological career aspirations were positively related to gender; but physics, mathematics, and engineering-related careers showed negative relationships. For dimensions of psychological wellbeing (depression, anxiety, and stress), gender positively related to stress (girls higher).

#### Achievement Background

Grade 9 mathematics (nationally assessed NAPLAN score) and science (self-reported ‘usual’ mark) achievement backgrounds were strongly positively associated. Mathematics achievement background significantly associated with all mathematics motivation factors: positively with perceived talent, intrinsic and utility values; negatively with all costs. Mathematics achievement background was unrelated to mathematics classroom learning environments. Students with higher mathematics achievement background aimed for substantially higher marks, exerted more effort, aspired to higher STEM careers, and reported lower depression, anxiety, and stress. Similarly, for science, achievement background was positively related to perceived talent, intrinsic, and utility values, and negatively related to costs. Science achievement background positively related to mastery-, and negatively to performance-oriented learning environments. Science achievement positively associated with aimed marks, effort exertion, STEM career aspirations, and lower levels of depression and anxiety, but not stress.

#### Motivation Factors

Among mathematics motivation factors, there were strong positive correlations between perceived talent, intrinsic, and utility values. The new cost factors negatively related to those positive motivations (social cost only related to utility value). The cost factors were moderately positively intercorrelated. Similarly, for science, there were strong positive correlations among perceived talent, intrinsic, and utility values. Cost factors were inversely related to these, and positively intercorrelated themselves. Between mathematics and science, positive motivation factors (perceived talent, intrinsic, and utility values) were all positively, moderately intercorrelated. The new cost factors also positively intercorrelated – strongly between corresponding mathematics and science costs, moderately between others.

#### Motivations, Achievement Striving, and Career Aspirations

Within each of mathematics and science, positive motivation factors (perceived talent, intrinsic, and utility values) were positively associated with aimed marks, effort exertion and STEM career aspirations; cost factors were negatively associated (social cost weakly negatively correlated only with subjectively rated science career and objectively scored biology career). In each of mathematics and science, higher achievement striving (aimed marks and effort exerted) related to STEM career aspirations. Subjectively rated mathematics and science career aspirations were moderately positively correlated. Objectively scored STEM career aspiration dimensions were highly correlated for biology and chemistry; also strongly among physics, engineering, mathematics, and chemistry; weakly between biology and others.

#### Experienced Learning Environments

Experienced mathematics mastery-oriented learning environment positively related to mathematics achievement striving; performance-oriented environments negatively related to aimed marks. Mastery environments positively related to all three positive motivations; and, negatively related to effort and psychological costs (unrelated for social cost). Conversely, mathematics performance environments negatively associated with intrinsic and utility values; positively with effort and social costs (unrelated for psychological cost). Mathematics mastery environments positively related to STEM-related career aspirations (except biology), whereas performance environments showed a mixed pattern of relationships.

In science, mastery environments positively related to achievement striving; performance environments negatively related to aimed marks. Science mastery environments positively related to all positive science motivations; negatively with all costs. Science performance-oriented environments weakly positively associated with perceived talent, negatively with utility value, and positively with effort and social costs. Science mastery environments positively related to subjectively rated science career aspirations and objectively scored chemistry, physics and mathematics careers. Science performance environments negatively associated with subjectively rated science careers and objectively scored biology and chemistry careers.

#### Psychological Wellbeing

Depression, anxiety, and stress were highly positively intercorrelated. Mathematics intrinsic and utility values were negatively associated with depression; perceived talent negatively associated with depression and anxiety. Depression negatively associated with mathematics aimed marks and effort exertion. The three mathematics costs positively (moderately) associated with depression, anxiety, and stress. In science, perceived talent and intrinsic value (but not utility value) negatively associated with depression and anxiety; perceived talent additionally negatively associated with stress. Aimed marks in science negatively associated with depression and anxiety; effort exertion associated with reduced anxiety. Science costs positively associated with depression, anxiety, and stress. Students who scored higher on depression aspired to less subjectively rated science careers and objectively scored physics and mathematics careers. Students who scored higher on anxiety aspired to lower subjectively rated science careers. Mastery learning environments exhibited a weak negative association with depression; science mastery environments additionally associated with reduced anxiety. Only science performance environments (not mastery) associated with higher depression, anxiety, and stress.

### Motivational Profiles

To move beyond patterns of association between variables and achieve our aim of identifying person-centred patterns, latent profile analysis (LPA) discerned three profiles in each of mathematics and science, based on the range of fit indices reported in Table 8. The profiles were named “Positively engaged” (high scores on the 3 positive motivation factors, low on the 3 negative costs), “Disengaged” (low on perceived talent and intrinsic value, high utility value and costs), and “Struggling ambitious” (high scores both on positive and negative factors). For mathematics, 638 students were Positively engaged (54.8%), 169 were Struggling ambitious (14.5%) and 357 were Disengaged (30.7%). In science, 726 students were Positively engaged (62.9%), 186 were Struggling ambitious (16.1%) and 242 were Disengaged (21.0%). Mathematics and science profile memberships were significantly associated, χ^2^(*N* = 1147, df = 4) = 306.365, *p* < 0.001. However, sizeable off-diagonal numbers indicated a substantial degree of non-overlap (i.e., domain specificity), most pronounced between Struggling ambitious and Disengaged profiles.

**TABLE 8:** Model fit indices for 1—5 latent profiles.

	**Number of latent profiles**				
	**1**	**2**	**3**	**4**	**5**
**Mathematics**					
AIC	24528.45	23341.18	22781.97	22495.83	22349.24
BIC	24589.16	23437.31	22913.52	22662.80	22551.63
aBIC	24551.05	23376.96	22830.94	22557.98	22424.57
Entropy		0.774	0.841	0.782	0.813
aLMR		1177.442	561.837	294.189	157.406
*p* aLMR		0.000	0.013	0.024	0.193
VLMR		–12252.20	–11651.60	–11365.00	–11214.90
*p* VLMR		0.000	0.012	0.022	0.187
**Science**					
AIC	24720.41	23111.20	22509.57	22133.62	21850.41
BIC	24781.02	23207.17	22640.90	22300.30	22052.45
aBIC	24742.90	23146.82	22558.32	22195.48	21925.40
Entropy		0.843	0.889	0.815	0.849
aLMR		1590.968	603.403	382.215	291.302
*p* aLMR		0.000	0.001	0.017	0.006
VLMR		–12348.20	–11536.60	–11228.80	–11033.80
*p* VLMR		0.000	0.000	0.016	0.006

Profile memberships differed by gender as anticipated. In mathematics, boys were overrepresented among the Struggling ambitious (20.5% boys versus 10.5% girls), and girls among the Disengaged (22.1% boys versus 36.4% girls), χ^2^(*N* = 1164, df = 2) = 39.397, *p* < 0.001. In science, boys were again overrepresented among Struggling ambitious (22.9% boys versus 11.5% girls), but girls among the Positively engaged (57.6% boys versus 66.5% girls), χ^2^(*N* = 1154, df = 2) = 26.574, *p* < 0.001.

Profiles were associated with home language background (English versus other), both in mathematics [χ^2^(*N* = 1123, df = 2) = 27.249, *p* < 0.05] and science [χ^2^(*N* = 1110, df = 2) = 8.766, *p* < 0.05], although patterns of association differed by domain. In mathematics, non-English language background (NELB) students were overrepresented among the Positively engaged (62.7% NELB versus 51.1% ELB) and English native speakers among the Disengaged (20.4% NELB versus 35.6% ELB). In science, NELB students were overrepresented among the Struggling ambitious (19.8% NELB versus 13.9% ELB) and English native speakers again among the Disengaged (17.4% NELB versus 22.6% ELB).

Parents’ levels of education were associated with profile memberships. In mathematics only father’s education background associated with student profiles, χ^2^(*N* = 1016, df = 6) = 12.700, *p* = 0.048. In science, associations were significant both for fathers, χ^2^(*N* = 1009, df = 6) = 15.346, *p* < 0.05, and mothers, χ^2^(*N* = 1067, df = 2) = 25.287, *p* < 0.05. In all cases, tertiary parent education levels (i.e., university or TAFE) associated with more Positively engaged profiles. Students whose mothers/fathers had not completed high school were overrepresented in Struggling ambitious profiles, and those whose parents had completed high school as their highest level of education were overrepresented among the Disengaged. Table 9 summarises demographic characteristics for each profile, in mathematics and science.

**TABLE 9:** Latent profile summary demographic characteristics.

	**Mathematics**							**Science**						
	**Positively**		**Struggling**					**Positively**		**Struggling**				
	**engaged**		**ambitious**		**Disengaged**			**engaged**		**ambitious**		**Disengaged**		
	***n***	**%**	***n***	**%**	***n***	**%**	**Total**	***n***	**%**	***n***	**%**	***n***	**%**	**Total**
**Mathematics strand (Melbourne)**														
General	343	52.77	93	14.31	214	32.92	650	–	–	–	–	–	–	–
Methods	112	77.78	19	13.19	13	9.03	144	–	–	–	–	–	–	–
**Mathematics strand (Sydney)**														
Standard	0	0	5	41.67	7	58.33	12	–	–	–	–	–	–	–
Intermediate	35	28.46	19	15.45	69	56.10	123	–	–	–	–	–	–	–
Advanced	130	64.68	29	14.43	42	20.90	201	–	–	–	–	–	–	–
**^a^Mother highest education**														
Part high school	67	49.63	23	17.04	45	33.33	135	76	56.30	29	21.48	30	22.22	135
High school	113	51.36	37	16.82	70	31.82	220	120	55.30	47	21.66	50	23.04	217
TAFE	63	57.27	39	35.45	8	7.27	110	76	70.37	8	7.41	24	22.22	108
University	360	59.11	77	12.64	172	28.24	609	418	68.86	77	12.69	112	18.45	607
**Father highest education**														
Part high school	55	46.61	21	17.80	42	35.59	118	76	65.52	24	20.69	16	13.79	116
High school	91	48.66	26	13.90	70	37.43	187	104	55.91	37	19.89	45	24.19	186
TAFE	47	56.62	9	10.84	27	32.53	83	55	66.27	10	12.05	18	21.69	83
University	372	59.24	83	13.22	173	27.55	628	422	67.63	79	12.66	123	19.71	624
**Selective schooling**														
Selective	283	69.70	36	8.87	87	21.43	406	325	79.85	26	6.39	56	13.76	407
Non-selective	355	46.83	133	17.55	270	35.62	758	401	53.68	160	21.42	186	24.90	747
**Home language**														
NELB	234	62.73	63	16.89	76	20.38	373	231	62.77	73	19.84	64	17.39	368
English	383	51.07	100	13.33	267	35.60	750	471	63.48	103	13.88	168	22.64	742

**^*a*^No significant association between mother’s education and mathematics profiles. All other associations were significant p < 0.05*.*

#### Achievement and Striving

##### Mathematics achievement background

Mathematics NAPLAN bands significantly differed according to mathematics profiles, *F*(2,858) = 57.35, *p* < 0.001 (all paired differences *p* < 0.01 in Tukey *post hoc* tests). Positively engaged students scored higher bands (*M* = 9.20, *SD* = 1.09), followed by Struggling ambitious (*M* = 8.66, *SD* = 1.37), and Disengaged scored lowest (*M* = 8.20, *SD* = 1.42). When selective school membership was added into the model as a second fixed factor, the main effect of mathematics profile remained significant, *F*(2,855) = 22.60, *p* < 0.001, along with a significant main effect of selective school attendance, *F*(1,858) = 150.25, *p* < 0.001. However, Bonferroni pairwise comparisons revealed that Positively engaged and Struggling ambitious profiles had equivalent NAPLAN achievement (*p* = 0.14) once selective school attendance was taken into account, although the other paired comparisons remained significant (*p* < 0.05). Table 8 shows proportions of students in each profile, who attended selective schools: 69.7% of Positively engaged, 8.9% of Struggling ambitious, and 21.4% of Disengaged. Consequently, construct comparisons by mathematics profiles were conducted with, and without, NAPLAN achievement as a covariate, to understand the unique role of mathematics profile membership on achievement striving, career aspirations, psychological wellbeing, and experienced learning environments.

##### Science achievement background

For science, self-reported ‘usual’ grade 9 results differed by science profiles, *F*(2,1066) = 145.75, *p* < 0.001 (all paired differences *p* < 0.01 in Tukey *post hoc* tests). Positively engaged students reported higher usual scores (*M* = 7.58, *SD* = 2.11), followed by Struggling ambitious (*M* = 5.34, *SD* = 2.68), and lowest scores were reported for Disengaged (*M* = 4.75, *SD* = 2.95). Because science assessments may not be comparable across schools or even classes, we decided against controlling for usual reported science scores in subsequent science construct comparisons.

##### Aimed marks and effort exerted

Positively engaged students aimed for higher marks in mathematics than Struggling ambitious (Table 10A), but their aimed marks were equivalent once NAPLAN achievement was included as a covariate (Table 10B). Disengaged students aimed for lowest marks in mathematics. All profiles differed significantly on effort exerted – Positively engaged were highest, followed by Struggling ambitious, and Disengaged were lowest. For science, Positively engaged students reported highest aimed marks, followed by Struggling ambitious, and then Disengaged; the same pattern occurred for reported effort.

**TABLE 10A:** Mathematics and science latent profile descriptive statistics and significant differences.

	**Mathematics**									**Science**								
	**Positively**		**Struggling**							**Positively**		**Struggling**						
	**engaged**		**ambitious**		**Disengaged**		***F***	**df**	***p***	**engaged**		**ambitious**		**Disengaged**		***F***	**df**	***p***
	***M***	***SD***	***M***	***SD***	***M***	***SD***				***M***	***SD***	***M***	***SD***	***M***	***SD***			
Perc. talent	5.07	0.98	4.64	1.17	3.02	1.14	411.27	2,1106	<0.001	5.02	1.01	4.18	1.10	2.91	1.24	333.25	2,1094	<0.001
Intrinsic value	5.26	0.96	4.40	1.27	2.64	1.09	675.15	2,1106	<0.001	5.72	0.89	4.41	1.34	2.85	1.16	676.64	2,1094	<0.001
Utility value	5.86	0.95	5.31	1.14	4.27	1.39	213.76	2,1106	<0.001	5.89	0.96	4.72	1.27	3.66	1.40	362.08	2,1094	<0.001
Effort cost	2.56	1.03	$4.07{}^{a}$	1.20	$4.07{}^{a}$	1.20	256.07	2,1106	<0.001	2.44	1.07	$4.33^{a^{\prime }}$	1.12	$4.48^{a^{\prime }}$	1.38	373.93	2,1094	<0.001
Psych. cost	3.08	1.52	5.05	1.28	4.38	1.62	147.95	2,1106	<0.001	3.04	1.59	4.74	1.15	4.10	1.74	101.65	2,1094	<0.001
Social cost	1.43	0.61	4.31	1.01	1.66	0.85	967.53	2,1106	<0.001	1.39	0.62	4.40	0.97	1.60	0.80	1224.61	2,1094	<0.001
Achievement background	9.20	1.09	8.66	1.37	8.20	1.42	57.35	2,858	<0.001	7.58	2.11	5.34	2.68	4.75	2.95	145.75	2,1066	<0.001
Performance	$3.02{}^{a}$	1.68	3.76	1.66	$3.01{}^{a}$	1.54	14.60	2,1146	<0.001	$2.75^{a^{\prime }}$	1.54	3.95	1.55	$2.87^{a^{\prime }}$	1.62	42.30	2,1114	<0.001
Mastery	5.22	1.37	$4.89{}^{a}$	1.35	$4.60{}^{a}$	1.59	21.39	2,1146	<0.001	5.54	1.25	$4.89^{a^{\prime }}$	1.48	$4.66^{a^{\prime }}$	1.60	43.99	2,1114	<0.001
Mark-aimed	8.44	1.54	7.44	2.27	5.90	2.64	170.63	2,1145	<0.001	8.23	1.62	6.51	2.54	5.52	2.73	175.85	2,1110	<0.001
Effort exerted	5.49	1.07	5.04	1.27	4.33	1.43	100.72	2,1127	<0.001	5.53	1.01	4.70	1.25	3.69	1.40	234.89	2,1109	<0.001
STEM career (subjective)	4.48	1.55	3.96	1.76	2.33	1.44	215.34	2,1127	<0.001	5.33	1.65	3.54	1.91	2.21	1.47	337.78	2,1109	<0.001
STEM career (O^*^NET)^*^																		
Biology	$43.32{}^{a}$	33.87	$32.90{}^{a}$	32.86	$27.65{}^{a}$	29.40	3.07	2,773	0.047	45.38	33.908	$27.18^{a^{\prime }}$	30.19	$17.83^{a^{\prime }}$	20.69	30.18	2,773	<0.001
Chemistry	$39.71{}^{a}$	24.62	$30.55{}^{a}$	23.73	23.47	21.50	9.62	2,773	<0.001	39.76	24.94	$26.61^{a^{\prime }}$	20.89	$18.56^{a^{\prime }}$	17.71	29.62	2,773	<0.001
Physics	$34.73{}^{a}$	23.61	$27.62{}^{\mathrm{ab}}$	20.44	$22.21{}^{b}$	19.39	9.52	2,773	<0.001	33.79	23.68	$25.48^{a^{\prime }}$	18.59	$20.95^{a^{\prime }}$	18.65	12.72	2,773	<0.001
Mathematics	$60.77{}^{a}$	17.187	$55.41{}^{a}$	17.84	47.89	16.15	17.76	2,773	<0.001	$58.63^{a^{\prime }}$	17.75	$55.39^{a^{\prime }}$	15.93	48.68	17.70	7.15	2,773	0.001
Engineering	$36.35{}^{a}$	28.00	$32.53{}^{\mathrm{ab}}$	23.61	$26.63{}^{b}$	21.30	5.10	2,773	0.006	$34.20^{a^{\prime }}$	27.43	$31.42^{a^{\prime }}$	22.37	$29.83^{a^{\prime }}$	22.83	0.99	2,773	0.373
Depression	0.68	0.60	1.16	0.79	0.94	0.77	34.76	2,1038	<0.001	0.70	0.63	1.20	0.82	0.92	0.71	38.84	2,1032	<0.001
Anxiety	0.57	0.55	0.99	0.78	0.75	0.72	27.98	2,1038	<0.001	$0.59^{a^{\prime }}$	0.58	1.05	0.82	$0.68^{a^{\prime }}$	0.62	34.92	2,1032	<0.001
Stress	0.80	0.68	1.16	0.82	0.95	0.76	16.11	2,1038	<0.001	$0.82^{a^{\prime }}$	0.71	1.16	0.85	$0.93^{a^{\prime }}$	0.68	14.57	2,1032	<0.001

^*a,b*^Paired letters within each row denote non-significant differences. All other means within each row significantly differ at p < 0.05.^*^O^*^NET scores were compared within a single MANOVA that included mathematics and science profiles as independent variables (n.s. interaction effect).

**TABLE 10B:** Mathematics latent profile descriptive statistics and significant differences – with NAPLAN covariate.

	**Positively engaged**		**Struggling ambitious**		**Disengaged**		***F***	**df**	***p***
	***M***	***SD***	***M***	***SD***	***M***	***SD***			
Perc. Talent	$5.12{}^{a}$	0.99	$4.79{}^{a}$	1.14	3.07	1.13	237.53	2, 817	<0.001
Intrinsic value	5.28	0.98	4.47	1.25	3.07	1.13	387.96	2, 817	<0.001
Utility value	5.82	0.96	5.32	1.15	4.30	1.39	130.62	2, 817	<0.001
Effort cost	2.51	1.01	$4.06{}^{a}$	1.23	$4.03{}^{a}$	1.20	153.77	2, 817	<0.001
Psych. cost	3.02	1.51	5.06	1.33	4.31	1.66	86.52	2, 817	<0.001
Social cost	1.41	0.61	4.38	1.04	1.61	0.81	703.97	2, 817	<0.001
Performance	$2.94{}^{a}$	1.64	3.65	1.72	$3.00{}^{a}$	1.55	8.06	2, 850	<0.001
Mastery	$5.18{}^{a}$	1.40	$4.79{}^{\mathrm{ab}}$	1.45	$4.55{}^{b}$	1.65	11.89	2, 850	<0.001
Mark-aimed	$8.51{}^{a}$	1.49	$7.85{}^{a}$	1.97	6.02	2.68	76.82	2, 849	<0.001
Effort exerted	5.54	1.07	5.04	1.36	4.35	1.41	67.16	2, 836	<0.001
STEM career (subjective)	4.51	1.54	3.89	1.79	2.36	1.49	123.10	2, 836	<0.001
STEM career (O^*^NET)^*^									
Biology	$44.34{}^{a}$	33.66	$37.68{}^{a}$	33.51	$29.98{}^{a}$	30.95	2.51	2, 576	0.082
Chemistry	$40.83{}^{a}$	25.10	$33.30{}^{a}$	24.65	24.38	20.95	7.13	2, 576	0.001
Physics	$34.15{}^{a}$	23.48	$30.03{}^{\mathrm{ab}}$	22.11	$21.80{}^{b}$	18.63	6.16	2, 576	0.002
Mathematics	$60.96{}^{a}$	17.22	$55.25{}^{\mathrm{ab}}$	19.03	$48.00{}^{b}$	15.44	12.70	2, 576	<0.001
Engineering	$35.36{}^{a}$	28.11	$33.11{}^{a}$	25.48	$26.42{}^{a}$	20.41	1.89	2, 576	0.152
Depression	0.67	0.60	1.19	0.76	0.94	0.77	25.28	2, 776	<0.001
Anxiety	0.55	0.54	1.01	0.76	0.78	0.72	20.42	2, 776	<0.001
Stress	$0.80{}^{a}$	0.69	1.19	0.82	$0.97{}^{a}$	0.76	11.35	2, 776	<0.001

^*a,b*^Paired letters within each row denote non-significant differences. All other means within each row significantly differ at p < 0.05.^*^O^*^NET scores were compared within a single MANOVA that included mathematics and science profiles as independent variables (n.s. interaction effect).

#### Perceived Classroom Learning Environment

Both in mathematics and science, Positively engaged students experienced classroom learning environments characterised by highest mastery orientation, whereas Struggling ambitious and Disengaged students scored similarly and lower. Conversely, Struggling ambitious students experienced the most performance focused classroom learning environments, whereas Positively engaged and Disengaged students scored similarly and lower (Table 10A). In mathematics, once NAPLAN achievement was included as a covariate to discern unique effects of mathematics profiles, Positively engaged students no longer differed from the Struggling ambitious on mastery environments (Table 10B).

#### Career Aspirations

For subjectively rated mathematics and science career plans, Positively engaged students aspired to careers more highly related to mathematics/science, respectively, followed by Struggling ambitious, and Disengaged students lowest (Table 10A). After controlling for NAPLAN achievement, the same pattern in subjectively rated mathematics careers remained (Table 10B).

For objectively coded STEM career aspirations, mathematics profiles differed on each of physics, chemistry, mathematics, and engineering careers (no significant differences for biology; Table 9). Specifically, Positively engaged and Struggling ambitious profiles were similar and highest for mathematics and chemistry career aspirations. They were also similar and higher on physics and engineering careers, although Struggling ambitious did not significantly differ from Disengaged. When NAPLAN achievement was included as a covariate (Table 10B), Positively engaged students had significantly higher mathematics, physics and chemistry-related career aspirations than Disengaged; however, there were no significant differences between profiles on biology or engineering. Struggling ambitious students scored similarly high to Positively engaged students on physics and mathematics career plans, but not significantly higher than Disengaged students. Struggling ambitious students were similarly high to Positively engaged students on chemistry career plans, and significantly higher than Disengaged.

Science profiles differed on each of objectively coded physics, chemistry, biology and mathematics career aspirations (no significant differences for engineering). Positively engaged and Struggling ambitious science profiles were similar and highest for mathematical career aspirations. Positively engaged science students were highest on the other STEM career dimensions, whereas Struggling ambitious and Disengaged students scored similarly and lower.

#### Psychological Wellbeing

Dimensions of psychological wellbeing were distinguished by profile memberships in each of mathematics and science. In mathematics, all profile differences were significant, with Struggling ambitious students highest on depression, anxiety, and stress, followed by Disengaged, and Positively engaged students lowest (Table 10A). With the NAPLAN achievement covariate included (Table 10B), Positively engaged and Disengaged profiles no longer differed on stress. In science, Struggling ambitious students were again highest on depression, anxiety, and stress. Positively engaged and Disengaged profiles were similar and lower on anxiety and stress; Disengaged students scored higher on depression (Table 10A).

## Discussion

This study followed two central aims. First, was to validate the new expectancy-value cost measure among adolescents across the domains of mathematics and science and examine associations with demographics, achievement background, mastery/ performance-focused learning environments, achievement striving, STEM-related career aspirations and dimensions of wellbeing. Second, was to discern hypothesised theoretically coherent expectancy-value latent profiles of students within each of mathematics and science, potential antecedents (demographics, experienced learning environments, and achievement background), outcomes (achievement striving, career aspirations, and psychological wellbeing), and degree of domain specificity versus generality.

### Expectancy-Value Cost Dimensions

Alongside typically examined positive motivational factors (i.e., perceived talent, intrinsic, and utility values), the purpose-adapted adolescent cost measure (based on Perez et al., 2014) proved well-functioning within each of mathematics and science and was key to identifying the student profiles to address our second aim. Effort, psychological and social costs were moderately positively intercorrelated within each domain (in contrast to extreme correlations between the dimensions by Flake et al., 2015), and were negatively correlated with domain-specific positive motivational factors, except social cost, which was not consistently significantly related. Between domains, the same cost factors were strongly correlated, and different costs were moderately correlated.

Psychological cost was highest rated (more so among girls, consistent with the mathematics anxiety literature), referring to concern about the degree of stress involved. Effort cost referred to the degree of effort required outweighing what students were willing to exert and social cost was least endorsed (intriguingly, higher among boys), tapping concern regarding loss of friendships due to working at mathematics/science. Students from more educationally advantaged backgrounds experienced lower costs. In general, performance-oriented learning environments related to increased effort and social costs in both domains, and mastery-oriented environments related to reduced costs (less so in mathematics). As we had anticipated, costs appeared to undermine achievement striving and STEM-related career aspirations (least so for social cost), and relate to heightened levels of general depression, anxiety and stress.

### Motivational Profiles in Mathematics and Science

Regarding the second aim and hypothesis, three profiles were supported in each of mathematics/science: the majority were Positively engaged (high on perceived talent and intrinsic/utility values), approximately one-sixth were Struggling ambitious (high on all), and 30.7%/21.0% (for mathematics/science, respectively) were Disengaged (low perceived talent and intrinsic value, high utility value and costs). The hypothesised Struggling ambitious type resonated with Covington and Omelich’s “overstrivers” (1991) and “excessively ambitious” workers in the German occupational health study (Schaarschmidt and Fischer, 1997). Although past studies found more boys to be represented in positive mathematics types (e.g., Chow and Salmela-Aro, 2011; Lazarides et al., 2019), such studies had not included costs in their profiling. With costs included, we found no gender difference in the positive mathematics type (and more girls in the positive science type), but a higher proportion of boys were split into the Struggling ambitious type both in mathematics and science. The Disengaged type enriched the mixed utility focused type identified by Lazarides et al. (2016a, b) in German and Finnish studies. As we had speculated, inconsistent valuing of mathematics/science as being useful, coupled with low interest value and perceived talent, coincided with elevated effort cost and psychological cost. Results were consistent with the research literature showing girls’ lower perceived talents and interest in mathematics (e.g., Watt et al., 2012) and higher mathematics anxiety (see Hyde, 2005); more girls fitted this type in mathematics. No gender difference was found in the Disengaged type for science, perhaps because physical and biological sciences were not distinguished, and girls tend to show more interest in biology.

Because mathematics involved a standard national assessment at grade 9, achievement background could be assessed on a common metric, unlike science. The achievement differences between mathematics profiles partly depended on selective school attendance – once accounted for, Positively engaged and Struggling ambitious profiles performed equally high in mathematics, aimed for similarly high marks, and aspired to similarly high objectively coded STEM careers. Struggling ambitious, however, reported most pronounced levels of depression, anxiety and stress. Although Positively engaged and Struggling ambitious experienced similar mastery-oriented learning environments, the Struggling ambitious students experienced more performance-focused environments. Disengaged students had significantly lower background mathematical achievement, exhibited lowest achievement striving, aimed for least STEM-related careers (except biology on which all profiles were similar), and scored in between the other two profiles on psychological wellbeing – equivalently low on stress as the Positively engaged profile once NAPLAN achievement background was controlled. Disengaged mathematics students came from similarly mastery-oriented learning environments as the Struggling ambitious, and similarly performance-oriented environments as the Positively engaged.

In science, Positively engaged students reported highest ‘usual’ achievement background, exerted highest achievement striving, aspired to highly STEM-related careers in biology, chemistry and physics (similarly high as the Struggling ambitious for mathematics careers; no profile differences for engineering), exhibited lowest levels of depression, and similarly low anxiety and stress as the Disengaged. Positively engaged science students came from the most mastery-oriented learning environments, and equally low performance-oriented environments as the Disengaged.

Overall, the Struggling ambitious profile (more boys) proved most maladaptive. In mathematics, despite equivalent achievement background, achievement striving and STEM-related career aspirations as a consequence of their high positive motivations, they suffered debilitated psychological wellbeing seemingly due to elevated costs, potentially exacerbated by their more performance-oriented learning environments. The Positively engaged profile was the most adaptive, exhibiting similarly high achievement striving and STEM career aspirations as the Struggling ambitious, and the most positive psychological wellbeing. The Disengaged profile (more girls) showed lowest achievement background, striving and career aspirations linked to their lower perceived talent and intrinsic value; and, moderate psychological wellbeing linked to their perceived effort and psychological costs.

In science, it was not possible to compare background achievement on any common metric, to disentangle profile effects for Struggling ambitious students who reported lower ‘usual’ marks, achievement striving, and some dimensions of STEM career aspirations than the Positively engaged. Struggling ambitious science students (more boys) reported the poorest psychological wellbeing, likely exacerbated by their more performance-oriented learning environments. In contrast, the Positively engaged profile experienced the highest mastery-oriented environments.

The typological approach moved beyond measuring differences in the extent of students’ motivations, to examine how they varied qualitatively in combination, enabling a more nuanced examination of complex relationships with potential antecedents and outcomes. Profiles related to past achievement and predicted mathematics/science achievement striving and career intentions, as well as psychological wellbeing. Profiles resonated with those educed using similar methods but different measures within other motivational frameworks and not specific to STEM. For example, our Positively engaged type shared features with the “mastery oriented” group among Finnish adolescents within an achievement goal perspective (Tuominen-Soini et al., 2012) and “engaged” group in another Finnish study among adolescents within a psychological wellbeing framework (highest school value, wellbeing and achievement; Tuominen-Soini and Salmela-Aro, 2014), and the “good motivated strategies for learning” group (positive motivation and achievement, low anxiety) in a study framed by self-determination theory (SDT) of Singaporean adolescents in mathematics and science (Ng et al., 2016).

Our Struggling ambitious type somewhat resembled the “success-oriented” group in the first Finnish study (Tuominen-Soini et al., 2012) who showed high motivation and achievement but emotional distress, the “engaged-exhausted” group in the Finnish psychological wellbeing study (high school value and achievement, but lower self-esteem, preoccupation with failures and depression; Tuominen-Soini and Salmela-Aro, 2014), and the “average motivation, low wellbeing” type in another Finnish study of adolescents in both mathematics and literacy (Parhiala et al., 2018). Similarities were also shown with the “high motivated strategies for learning” group (high motivations, moderate achievement, high anxiety) among Singaporean students (Ng et al., 2016), and “moderate engagement but maladaptive cognitions” in an Australian tertiary study (Elphinstone and Tinker, 2017).

Finally, our Disengaged type resembled the “cynical” group in the Finnish study (lowest school value and achievement, moderate wellbeing; Tuominen-Soini and Salmela-Aro, 2014) and the “poor motivated strategies for learning” group (lowest motivations and achievement, high anxiety) in the Singapore SDT study (Ng et al., 2016).

### Implications

The results of this study have theoretical implications. Our study has linked the breadth of expectancy-value constructs including multidimensional costs explicitly to measures of experienced learning environments as conceptualized within achievement goal theory, psychological wellbeing, achievement striving and career aspirations, in relation to two key domains of mathematics and science. Including negative cost values alongside typically measured positive expectancy-value dimensions enabled identification of students who experience particular combinations of motivations and pressures, contrasting with variable-centred approaches where the focus is on normative patterns. Profiles could be conceptualized along dimensions of achievement striving and psychological wellbeing. Similar profiles for mathematics and science, and coherent patterns of antecedents and outcomes, suggest they deserve further investigation. Positively engaged/Struggling ambitious were distinguished by high costs perceived by Struggling ambitious, associated with debilitated psychological wellbeing, but not eroding achievement striving. A greater proportion of boys was in this risk type. Disengaged students reported lowest STEM-related career aspirations, aimed marks, and history of results; in mathematics, a greater proportion of girls was in this risk type. Gender differences may be reflective of the pressures and expectations resulting from entrenched stereotypes that boys should be naturally better in STEM subjects – more boys were Struggling ambitious (in mathematics and science), and that girls are not expected to be good at or interested in mathematics – more girls were Disengaged (in mathematics).

This study provides some practical implications, as well. The fact that there were differences in students’ memberships across mathematics/science profiles suggests that while there may be a dispositional or core base, there is much shaped within each learning domain. These differences signal amenability to change by intervention (Crick, 2012). Eccles and her colleagues have demonstrated that girls are engaged by activities they perceive as socially meaningful and important (Watt et al., 2012; Eccles, 2013), but because mathematics is frequently taught in skills-based, abstract, decontextualised ways, it would seem less likely to engage girls. Practical approaches could include making explicit connections between mathematics and its social uses and purposes, to especially help girls who were overrepresented in the Disengaged profile to develop a sense of personal significance and practical value (Su et al., 2009; Eccles and Wang, 2012; Watt et al., 2012). For example, a promising utility-value intervention for parents has shown positive effects on STEM career preparation and pursuit (Rozek et al., 2017). Our findings suggest that the learning environment may also be a potential target of intervention.

Situating students’ motivational profiles within the ecology of their experienced learning environments revealed systematic differences across examined mastery- and performance-oriented dimensions. A focus on developing mastery-oriented classrooms could be the starting point to promote students’ engagement in STEM through the nature of the motivational environments that teachers create. Distinct profiles of motivation within classrooms suggest that teachers may need to tailor approaches for the different types, keeping in mind that even within subgroups, one size does not fit all (Lawson and Lawson, 2013). Of particular concern is what may happen to the Struggling ambitious students whose experienced learning environments related to heightened experiences of depression, anxiety, and stress. Relatedly, for the “engaged-exhausted” profile identified among Finnish students (Tuominen-Soini and Salmela-Aro, 2014), authors recommended a learning environment that does not focus on performance or social comparison, and cautioned the danger of overlooking these students because of their positive academic motivation and achievement. Covington (1992) similarly expressed concern that the failure avoidance and success of “overstrivers” can come at great cost to their wellbeing and highlighted that risk as a core issue that should be of concern to researchers. In future, it will be important to discover whether Struggling ambitious students adapt their standards and expectations over time and shift to one of the other profiles, or become exhausted, unable to cope and eventually burn out.

### Limitations

In interpreting the results, some limitations should be kept in mind. First, the study was limited to grade 10 Australian students from nine metropolitan schools in Melbourne and Sydney, some of which had rather low response rates. It would be very interesting to see if the result patterns can be replicated with older or younger students, and how profile memberships may change over time. Second, this study relied on student-reported achievement assessments in science, which was not comparable across classes or schools. Third, with the exception of background achievement information, data were cross-sectional, and longitudinal or intervention studies would be required to tease apart directionality of effects between profiles and correlates. Finally, this study is limited to the domains of mathematics and science; science in particular is a multifaceted field which could be fruitfully disaggregated into its component disciplines, as especially biology showed different effect patterns in terms of career aspirations.

### Conclusion and Outlook

Although similar profiles were educed within each of the cognate domains of mathematics and science, the fact that substantial numbers of students were in different profiles for mathematics versus science supports a domain-specific interpretation. Patterns of correlation for the same constructs across domains strengthens this inference. Student characteristics interacted with features of their learning environments with implications for their achievement striving, career aspirations and psychological wellbeing. This study leads to some intriguing future questions, most importantly, how to shift students to a more adaptive profile? While it was encouraging to observe that most students were characterised as “positively engaged”, they may not remain so, especially in unsupportive learning environments. It is also of high concern that Struggling ambitious students suffered elevated costs and debilitated psychological wellbeing, and that sizeable proportions were Disengaged. This implies the need for a dual focus in the design of productive interventions to harness conditions that facilitate and sustain positive motivations, at the same time as providing support for students experiencing anxiety or difficulties. Our study has contributed to the expectancy-value body of literature by furthering our understanding of how dimensions of students’ expectancies and values, including costs, combine, and their importance for a range of academic, vocational, and psychological outcomes along the STEM pipeline.

## Ethics Statement

This study was carried out in accordance with the recommendations of the Monash University Human Research Ethics Committee (MUHREC) with written informed consent from all participants in accordance with the MUHREC requirements. The project was approved by MUHREC, the Department of Education and Early Childhood Development (DEECD), NSW Government Education & Communities State Education Research Application Process (SERAP), and the Catholic Education Office Melbourne (CEOM).

## Author Contributions

All authors listed have made a direct and intellectual contribution to the work, and approved it for publication.

## Conflict of Interest Statement

The authors declare that the research was conducted in the absence of any commercial or financial relationships that could be construed as a potential conflict of interest.

## Footnotes

1 www.stepsstudy.org

2 In actuality, only 2 students were differently classified (in the mathematics LPA) when single-level analyses were conducted.
